# The effect of perinatal interventions on parent anxiety, infant socio‐emotional development and parent‐infant relationship outcomes: A systematic review

**DOI:** 10.1002/jcv2.12116

**Published:** 2022-11-19

**Authors:** Celia G. Smith, Emily J. H. Jones, Sam V. Wass, Dean Jacobs, Cassie Fitzpatrick, Tony Charman

**Affiliations:** ^1^ Institute of Psychiatry, Psychology & Neuroscience King's College London London UK; ^2^ Birkbeck University of London London UK; ^3^ University of East London London UK

**Keywords:** dyadic mechanisms of psychopathology, infant mental health, infant socio‐emotional development, parent‐infant relationship, perinatal anxiety

## Abstract

**Background:**

Infants of parents with perinatal anxiety are at elevated likelihood of experiencing disruption in the parent‐infant relationship, as well as difficulties with socio‐emotional functioning in later development. Interventions delivered in the perinatal period have the potential to protect the early dyadic relationship and support infants’ ongoing development and socio‐emotional outcomes. This review primarily aimed to examine the efficacy of perinatal interventions on parent anxiety, infant socio‐emotional development/temperament, and parent‐infant relationship outcomes. Secondarily, the review sought to understand how interventions focused principally on one member of the dyad affected the outcomes of the other, and which intervention components were common to successful interventions.

**Method:**

Five electronic databases as well as manual search procedures were used to identify randomised controlled trials according to a PICO eligibility criteria framework. Risk of bias assessments were undertaken, and a narrative synthesis was conducted. The review was pre‐registered on PROSPERO (CRD42021254799).

**Results:**

Twelve studies were analysed in total, including five interventions focused on the adult, and seven interventions focused on the infant, or the infant’s relationship with their parent. Interventions incorporating cognitive behavioural strategies for affective disorders showed reductions in parent anxiety (*N* = 3), and interventions focusing on altering distorted maternal internal representations showed positive change in parent‐child dyadic interactions, and infant outcomes (*N* = 2). Evidence that interventions focused on one partner of the dyad led to improved outcomes for the other partner was limited. However, evidence was of mixed methodological quality.

**Conclusions:**

It is important to integrate both parents and infants into treatment programmes for perinatal anxiety. Implications for clinical practice and future intervention trials are discussed.

**Key points**

- Perinatal anxiety associates with adverse parental outcomes, as well as infant socio‐emotional difficulties and alterations in the parent‐infant relationship.
- Interventions incorporating cognitive behavioural strategies demonstrate improvements in parent anxiety outcomes during the perinatal period.
- Perinatal interventions focusing only on the parent's anxiety tend not to demonstrate improvements in infant or parent‐infant relationship outcomes.
- Interventions addressing distorted maternal representations, and emphasising the infant's uniqueness/individual agency, may facilitate improvements in the parent‐infant relationship or infant socio‐emotional functioning.
- By combining (1) interventions targeting parent‐infant interaction dynamics and (2) cognitive behavioural interventions for parents, perinatal anxiety treatment has the potential to improve outcomes for both parents and children.

## INTRODUCTION

### The relationship of perinatal anxiety to infant and parent‐infant outcomes

Perinatal anxiety refers to a mental health condition characterised by cognitive distortions, physiological arousal, and behavioural avoidance; these are experienced either in the prenatal period, or in the immediate year after birth (Harrison & Alderdice, 2020). Due to high point prevalence rates of approximately 15%, perinatal anxiety has become recognised as a prominent public health issue (Dennis et al., 2017; Leach et al., 2017). The condition has been associated with numerous adverse maternal and neonatal outcomes, including maladaptive maternal coping strategies (e.g., self‐blame, denial; George et al., 2013), maternal suicidality (Farias et al., 2013), birth complications (Dowse et al., 2020), preterm birth, low birth weight (Ding et al., 2014) and fear of childbirth (both an anxiety condition in its own right and a possible outcome of other anxiety presentations; Demšar et al., 2018). In addition, perinatal anxiety has been associated with a range of negative consequences for later child development (O’Connor et al., 2002; O’Donnell et al., 2014; Polte et al., 2019; Rees et al., 2019) as well as perturbations in the parent‐child relationship (Feldman, 2007; Murray et al., 2008; Smith, 2022).

Perinatal anxiety is known to alter the early parent‐infant relationship. Higher maternal state anxiety is associated with lower levels of sensitive behaviour during mother‐infant interactions at 3 months (where sensitivity is defined as parental responsivity to infant activities and affective states; Ierardi et al., 2019). This is important as insensitive parental behaviour plays a causal role in shaping insecure child attachment (Bakermans‐Kranenburg et al., 2003). In addition, when compared to ‘healthy’ adults and their infant partners, anxious parents exhibit more frequent parental expressions (e.g., infant‐directed speech and positive facial expressions; Murray et al., 2008; Granat et al., 2017), higher unpredictability (i.e., inconsistency in the order of parental sensory signals; Holmberg et al., 2020), increased intrusive behaviour (overcontrolling behaviour that restricts child autonomy; Hakanen et al., 2019), and highly synchronous parent‐infant behaviour (Beebe et al., 2011; Granat et al., 2017). Anxious parents also show higher physiological synchrony with their infants, driven by higher reactivity to small‐scale fluctuations in infant arousal (Smith et al., 2021); and anxious caregivers are more likely to vocalise in clusters (i.e., aperiodic ‘bursts’ followed by lulls of inactivity; Abney et al., 2018) to their child at times when their own physiological arousal is elevated (Smith et al., 2022).

There is further evidence from experimental and longitudinal studies that perinatal anxiety associates with atypical infant socio‐emotional development. A recent prospective study of mothers with and without perinatal anxiety found that perinatal anxiety significantly increased the odds of difficulties in their two‐year‐old's socio‐emotional functioning, such as self‐regulation, by a factor of four (Polte et al., 2019), equivalent to a large Cohen's *d* effect size (Chen et al., 2010). This finding is consistent with evidence indicating that perinatal anxiety relates to early signs of avoidant behaviour in children (e.g., hiding from, ignoring, or looking/turning away from interaction; Aktar et al., 2013a; Murray et al., 2008), atypical social information processing in children (e.g., aversion or bias to facial expressions of fear; Creswell et al., 2008, 2011), and increased likelihood of childhood anxiety disorders (Lawrence et al., 2020).

There is preliminary evidence that perinatal interventions for anxiety have a positive effect on parent outcomes (Loughnan et al., 2018); however, there have been few studies in this area, and less still is known about the effect of interventions for perinatal anxiety on infants. Interventions have typically focused on only the adult member of the dyad (Loughnan et al., 2019; Maguire et al., 2018; Sockol, 2018). However, interventions that incorporate a focus on the infant or the dyadic relationship may serve to improve parent‐infant relationship dynamics and subsequent child outcomes. This view is coherent with the mutual regulation model, which holds that infant socio‐emotional function is fostered through dyadic, coregulatory behaviours (Tronick, 2007). Research suggests that perinatal mental illness interferes with this process through unresponsive, insensitive parental behaviour that leads to dysregulation of infants' affective states, even when interacting with others (Field et al., 1988; Weinberg & Tronick, 1998). Efforts to modify parental behaviour in perinatal interventions may therefore help promote coregulation, and improve child outcomes (Stein et al., 2014).

### Perinatal mental illness interventions and infant outcomes

To date, there have been no previous systematic reviews or meta‐analyses addressing how perinatal interventions relate to parent anxiety, the parent‐infant relationship and infant socio‐emotional development. This may be due in part to the historical emphasis on interventions for postnatal depression, which has been the focus of the vast majority of studies on perinatal mental illness over the past 30 years (Howard et al., 2014).

There have been numerous reviews on the efficacy of interventions for postnatal depression in relation to infant outcomes (Letourneau et al., 2017; Poobalan et al., 2007; Tsivos et al., 2015). The most recent review found little evidence for therapeutic effects (Rayce et al., 2020). It is also worth noting that perinatal anxiety frequently co‐occurs with depression (e.g., prevalence of a clinical diagnosis of any prenatal anxiety disorder and depression is 9.3%; Falah‐Hassani et al., 2017) and can worsen depression outcomes (Kalin, 2020). Yet none of these reviews extracted data on parent anxiety outcomes from the included studies (Letourneau et al., 2017; Poobalan et al., 2007; Tsivos et al., 2015).

One review of the effects of perinatal interventions on infant and dyadic outcomes has recently been conducted, examining parents with a broad range of perinatal mental health difficulties (Newton et al., 2020). This found that six interventions that supported with understanding the infant's perspective, as well as five interventions that incorporated video‐feedback and facilitation of mother‐infant interaction, were effective for infant and parent‐infant outcomes (e.g., secure attachment; increased parental sensitivity). These findings are consistent with a previous review examining 22 studies, which showed that video feedback improves parental sensitivity among young children at risk of poor attachment outcomes (though note, not specifically at risk of parental anxiety; O’Hara et al., 2019). Combined, these reviews suggest that infant‐focused perinatal interventions may be beneficial in a range of clinical contexts.

Despite these recent advances, there remains a gap in the intervention literature. Both Newton et al. (2020) and O’Hara et al. (2019) are broad in scope and do not provide a specific focus on perinatal anxiety or its particular developmental sequelae in children. In addition, Newton et al. (2020) includes numerous studies at high risk of bias due to lack of randomised allocation, and lack of masking among outcome assessors. O’Hara et al. (2019) also omits studies of multifactorial psychosocial interventions, that is, interventions including a range of components (e.g., cognitive behavioural therapy, which involves elements such as psychoeducation, cognitive techniques such as cognitive re‐structuring, and behavioural approaches such as graded exposure). Given that multifactorial parental interventions are the most widely available treatments within health systems, reviews evaluating these types of interventions are necessary. Finally, there have been a number of large studies in the recent period that focus on interventions for perinatal anxiety and infant outcomes, which have not been captured by extant reviews (e.g., Burger et al., 2020; Holt et al., 2021). Hence there is a need for a specific review of multifactorial perinatal interventions with respect to parent anxiety and infant outcomes.

### The present review

Considering that perinatal anxiety associates with atypical infant socio‐emotional development (Aktar & Bögels, 2017; Aktar et al., 2013b) as well as parent‐infant relationship perturbations (Feldman et al., 2009; Ierardi et al., 2019; Rees et al., 2019), it is important that we establish which perinatal anxiety interventions, if any, predict better outcomes for parents and infants. To address this, the following systematic review examines the efficacy of perinatal interventions on parent anxiety, infant socio‐emotional development or temperament, and parent‐infant relationship outcomes. Following the theoretical framework of Tronick (2007), we also explore how interventions focused predominantly on one member of the dyad (e.g., the adult) affected the outcomes of the other (e.g., infant temperament or socio‐emotional development). Finally, we take a mechanistic approach, exploring whether there are any common components among the interventions that demonstrate significant improvement in the outcomes of interest.

## METHOD

### Eligibility criteria

To review how perinatal interventions affect parent anxiety, infant socio‐emotional development, and parent‐infant relationship outcomes, we aimed to identify all peer reviewed papers on this topic. The review protocol was preregistered with the NIHR international prospective register of systematic reviews (PROSPERO; CRD42021254799). Studies were included if they met the following criteria:

(1) participants were pregnant people or parents (of any age or gender) of infants up to mean age of 24 months at study entry; parents were also identified to be at specific risk of or meet criteria for psychiatric disorders such as affective disorders, obsessive‐compulsive disorder (OCD), posttraumatic stress disorder (PTSD), or specific phobia (e.g., tokophobia);
(2) a psychosocial and/or pharmacological intervention was delivered either postnatally or a combination of pre‐ and postnatally; interventions delivered only prenatally, but with an infant follow‐up were also considered; group/individual/web/in‐person delivery formats of any duration were all acceptable;
(3) a control group was present, and participants were randomly allocated to either the control or the intervention group(s);
(4) parent anxiety was measured both pre‐ and post‐intervention by a continuous or categorical variable. One or more of the following infant outcome measures was also measured pre‐ and post‐intervention (or only post‐intervention if interventions were delivered exclusively in the prenatal period): infant socio‐emotional development, infant temperament, and parent‐infant bonding;
(5) studies conformed to randomised controlled trial standards, by use of randomisation procedures outlined in the CONSORT 2010 guidance (Schulz et al., 2010). No minimum sample size was required.

Studies were excluded if infant participants were exclusively preterm or cared for in neonatal intensive care units or if no randomised control group was present. Studies that did not conform to randomised controlled trial standards were also excluded.

To allow greater comparability and generalisation to clinical populations, the review included studies where: (a) samples were recruited on the basis of parent psychopathology; (b) the infant or dyadic outcome measures pertained specifically to infant rather than fetal phenomena, and (c) the parent outcome measure pertained to anxiety symptomatology or disorders, including disorders previously classified under the category of anxiety in diagnostic manuals (e.g., PTSD and OCD; Craske et al., 2017). Studies were therefore excluded if the sample was recruited on the basis of broad risk categories, such as economic disadvantage, transition to parenthood, infertility, or having a child with a behavioural problem or developmental condition. Studies were also excluded if the intervention or outcome was focused on parent psychopathology, but the recruitment was not. Further detail on population scoping is given in the SM (Section 1).

In addition, studies were excluded if the parent anxiety outcome was part of a broad mood measure (e.g., the self‐reporting questionnaire, SRQ‐20; Husain et al., 2016), or if the measure related to the construct of stress rather than anxiety per se. Due to specialist advice that methodological filtering by English language represents a ‘blunt tool,’ preventing the retrieval of eligible records, this was not part of the search strategy. Where possible we endeavoured to include publications in multiple languages (e.g., English, German). However, there was one occasion in which a study was reported in a language that was not machine‐translatable; this was due to the document not being ‘text mineable’ (i.e., text was presented as an image) and therefore this study was excluded.

### Search strategy

Both manual and electronic database searches were included in the search strategy. Manual searches included both hand searching and contact with key experts. Between 17th May and June 5th 2021, five electronic databases were searched via two interfaces: MEDLINE (via OvidSP), EMBASE (via OvidSP), APA PsychINFO (via OvidSP), MIDIRS (via OvidSP), and the Cochrane Central Register of Controlled Trials (via CENTRAL). Search terms were developed with guidance from an information specialist at King's College London and were optimised for each database. Electronic searches used MeSH and other subject headings as well as adjacent word searching and truncation. An expansive approach to field searching was taken (e.g. mp *v.* ti.ab) so as not to omit records that included key outcome measures in the main text but not the title or abstract. All search terms are detailed in the SM (Tables S1‐S5).

After the electronic searches were complete, manual searching was performed. For all included records, this involved reference list searching, whereby any titles that appeared relevant were identified by hand and subsequently retrieved. In addition, citation searching was performed using the citation search function on Google Scholar and the interactive infographic accompanying searches on Connected Papers. Recent guidance on the use of web search tools was followed (Briscoe et al., 2020). Finally, 12 key experts were contacted to identify any recent and eligible records (experts were senior authors of the included studies).

### Procedures

Retrieved records were downloaded into bibliographic software (Zotero Desktop Reference Manager, version 5.0.96.2). Duplicates were removed first through automation using the online web application Deduplicator (Rathbone et al., 2015) and then checked by hand by the lead author (CS). Two reviewers (CF, DJ) independently conducted title and abstract screening via the platform Screenatron (Clark et al., 2020; Scott et al., 2021), marking records as ‘Included’ if they met all the inclusion criteria and ‘Excluded’ if they did not. The review team also created a ‘Maybe’ category for records meeting all inclusion criteria except the parent anxiety outcome measure. This was due to a scoping exercise conducted prior to the review that indicated the high frequency with which secondary or tertiary anxiety measures tended to be omitted in the abstract but present in the full article. Accuracy measures were calculated on included records, and disputes between reviewers were identified using the online web application Disputatron (Clark et al., 2020; Scott et al., 2021). Disputed records were screened and reclassified by CS. Subsequently, all records marked included/maybe from the electronic search were screened at full text by CS. Records retrieved through manual searching were also screened at full text. The lead author's judgements were verified through discussion with the review team, which involved approximately 10% of full texts being rescreened.

### Data extraction and risk of bias assessments

The Cochrane Collaboration data extraction form for randomised controlled trials (Cochrane Collaboration, 2014) was used across all eligible studies. To ensure our review represented the latest developments in quality assessment, risk of bias (RoB) assessments were conducted using the Cochrane Collaboration's RoB Tool (Sterne et al., 2019). The updated tool marks a departure from earlier versions based on subjective ratings across broad domains of bias (selection bias, performance bias, attrition bias, and reporting bias; Higgins et al., 2011). Instead, algorithmically informed bias assessments are conducted across five more specific domains: bias arising from the randomisation process, bias due to deviations from the intended intervention, bias due to missing outcome data, bias in measurement of the outcome, and bias in selection of the reported result. Cochrane Collaboration's macro‐enabled Microsoft Excel tool was used to perform structured assessments (RoB 2, version 22 Aug 2019). Fifty percent of the bias assessments were also performed independently by a separate reviewer (DJ) to identify any discrepancies and reach consensus judgements. The results were plotted using the Robvis tool due to good interoperability with the Excel tool (McGuinness & Higgins, 2020).

### Analysis

Using an approach adapted from a previous review of perinatal interventions, components of interventions from the included studies were extracted and tabulated to ‘develop a matrix mapping the key components of the studies against the study results’ (Newton et al., 2020, p. 3). The matrix was split according to whether the intervention predominantly focused on the parent or the infant/dyad (of note, dyadic outcomes were grouped together with ‘infant outcomes’ due to strong associations between the parent‐infant relationship and infant socio‐emotional development; Feldman & Eidelman, 2004; Feldman, 2007; Feldman, 2021). This allowed for an examination of whether there were ‘symmetrical’ effects (adult‐focused interventions that led to improved parent outcomes, and infant/dyad‐focused interventions that led to improved infant/dyad outcomes) and ‘asymmetrical’ effects (infant/dyad‐focused interventions that led to improved parent outcomes, and adult‐focused interventions that led to improved infant/dyadic outcomes). In order to facilitate a consideration of the mechanisms of treatment outcomes, we also used the intervention components matrix to identify any components common to interventions that demonstrated significant improvements in the outcomes of interest. We elected not to perform a meta‐analysis due to the high level of heterogeneity among the infant outcome measures.

For four studies, deviations from the intended intervention were identified from inspecting trial registry records, trial protocols and journal articles for each study. For the purposes of being consistent and precise, the decision was taken to restrict the component analysis to the information available in the journal article and trial protocol. These documents are more contemporaneous with one another than the trial registry record, and more comprehensive. To mitigate bias toward interventions familiar to the lead author, the final intervention component list was discussed and agreed by the full review team.

## RESULTS

### Search results

A total of 2070 records were retrieved from electronic searches. Before title and abstract screening, 318 duplicate records were excluded, with 1752 records remaining. Accuracy measures calculated from title and abstract screening indicated high inter‐rater reliability between two independent reviewers (DJ and CF screened all 1752 records; *κ* = 0.78; prevalence and bias adjusted kappa [pabak] = 0.98). Subsequently, 1585 records were excluded due to ineligibility and 167 records were retrieved for full text screening. Of these, the following records were excluded: 95 records reporting no specific parent anxiety outcome at pre/post‐intervention, one featuring no relevant infant/dyad outcome, 27 featuring child participants who were too old, and 18 featuring samples that were not recruited on the basis of parent psychopathology. We also excluded: 10 conference abstracts, five duplicates not previously identified due to inconsistent metadata, and one record written in a language not spoken by the review team. One record was also excluded due to unreliable reporting indicated by numerous inconsistencies in the manuscript (including those pertaining to the main findings, outcome measures, and intervention description).

A total of 16 records were also retrieved from manual searching. Full texts of these were inspected and the following exclusions were made: four records for which there was no specific parent anxiety measure reported at pre/post‐intervention; two records featuring no relevant infant/dyad outcome; four records for which parent psychopathology was not the focus of recruitment; two records featuring child participants who were too old, and one record that had not been peer reviewed (an unpublished thesis).

**FIGURE 1:**
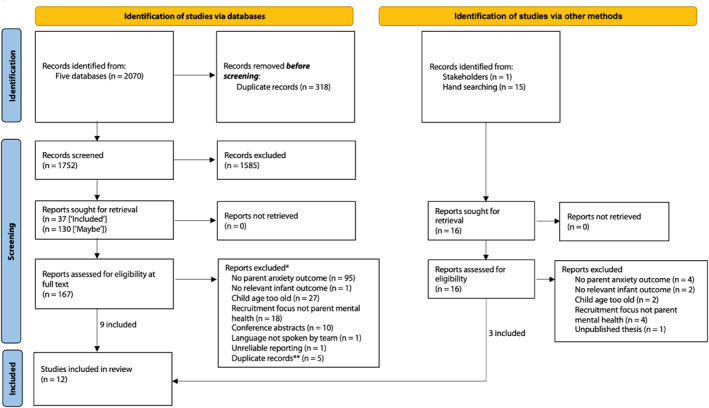
PRISMA flow diagram. Note that hand searching comprises both citation and reference searching. *This list represents one failed inclusion criterion per study—however, multiple studies failed to meet more than one inclusion criteria, as detailed in Table S6. **These records had not been previously identified due to inconsistencies between database metadata. Adapted from Page et al. (2021)

Consequently, 12 studies were included in the final review, including nine from the electronic search and three from the manual search. Figure 1 details the full screening results in a PRISMA flow diagram. In addition, reasons for exclusion and inclusion of all studies screened at full text are detailed in Tables S6‐S7 respectively (SM).

### Risk of bias assessments

**FIGURE 2:**
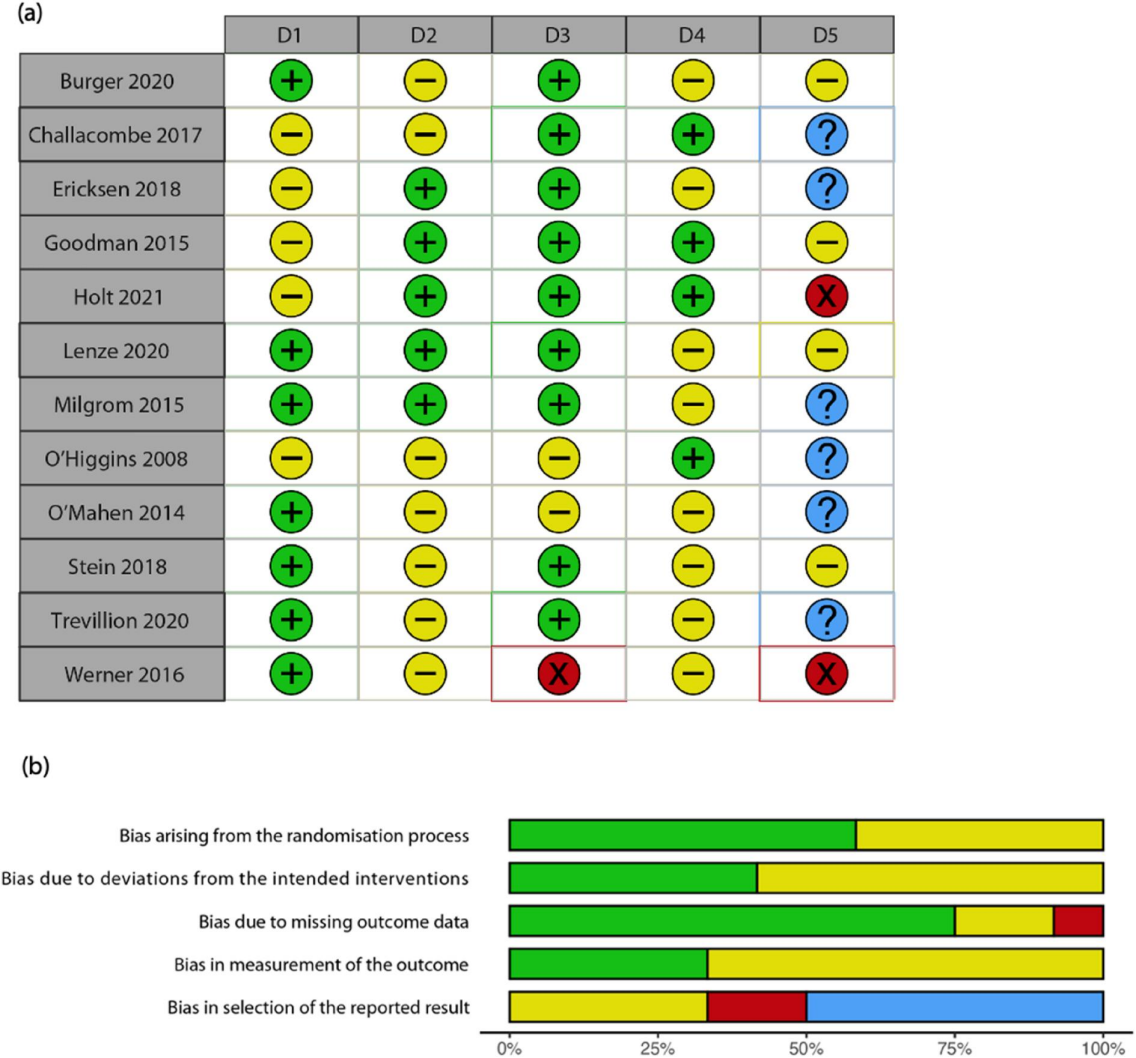
(A) Traffic light plot summarising Cochrane risk of bias assessments; D1—bias arising from the randomisation process; D2—bias due to deviations from the intended intervention; D3—bias due to missing outcome data; D4—bias in measurement of the outcome; D5—bias in selection of the reported result; (B) summary plot aggregating the bias assessment results across the 12 studies for the five listed domains. Colours: red—high risk of bias; yellow—some concerns; green—low risk of bias; blue—no or inadequate information available for assessing intended analyses

An overview of the results from the risk of bias assessments is presented in Figure 2. Further details of the assessments are given in the SM.

### Study characteristics

Twelve studies involving 1029 participants were included in the review in total; key study characteristics are presented in Table 1. Half of the studies were published within the last three years, with the remainder spanning the period between 2008 and 2017. All the studies' adult participants were women of working adult age, and all infants were under the age of 7 months at study entry. In four studies, 33%–80% of participants were from minoritised ethnic backgrounds, while the remaining eight studies' samples consisted of those from white, majority ethnic backgrounds. There was variation across the studies with respect to inclusion criteria for adult psychiatric risk (Table 2), as well as outcome measures for parent anxiety, infant socio‐emotional development, and the parent‐infant relationship (Table 1); there were also differences across studies in terms of intervention components (Table 3).

**TABLE 1:** Participant characteristics including age of both parent and infant, as well as parent anxiety level, and ethnicity; collected at baseline across all studies

	*N*	Mean (SD) parent age		Mean (SD) infant age in postpartum mos unless specified		Mean (SD) anxiety score (or % with diagnosis)		Parent anxiety measure	Black and ethnic minority (including ‘other’) %	
		Intervention	Control	Intervention	Control	Intervention	Control		Intervention	Control
Burger 2020	282	33.4 (4.6)	32.1 (4.5)	3.5 gestation		48.6 (8.7)	48.5 (8.4)	Brief STAI^1^	6.0	2.2
Challacombe 2017	71	32.4 (no SD)	32.7 (no SD)	∼6		24.82 (5.19)	24.47 (5.81)	YBOCS^2^	18	12
Ericksen 2018	31	32.31 (6.04)	33.00 (6.38)	4.94 (2.91)	4.87 (1.81)	17.25 (no SD)	14.67 (no SD)	DASS anxiety^3^	Not reported	
Goodman 2015	42	30.57 (4.760)	30.81 (5.316)	Not reported		43.62 (9.47)	36.00 (10.39)	STAI‐S^4^	42.9	38.1
Holt 2021	77	32.13 (5.04)	33.33 (3.85)	3.13 (2.67)	3.97 (2.87)	15.4 (9.29)	13.66 (7.35)	BAI^5^	Not reported	
Lenze 2020*	42	26.90 (5.81)	26.38 (5.90)	∼3‐7.5 gestation		15.6 (6.5)	15.0 (4.2)	Brief STAI‐S^6^	81	86
Milgrom 2015	54	32.79 (5.97)	30.78 (5.86)	4.99 gestation	5.24 gestation	22.37 (10.05)	20.59 (10.67)	BAI^5^	Not reported	
O’Higgins 2008	96	Not reported		∼2.5		44.7 (11.25)	45.49 (12.84)	STAI‐S^4^	Approx. 30	
O’Mahen 2014	83	Not reported (except: >18)		Not reported (except: <12 mos)		13.90 (3.82)	14.12 (4.78)	GAD‐7^7^	7.2	7.2
Stein 2018	144	31.7 (5.7)	32.2 (5.3)	6.8 (2.0)	6.8 (1.9)	48.6%	32%	SCID‐IV‐R^8^	15.3	19.4
Trevillion 2020	53	30‐39 (∼69%)	30‐39 (∼67%)	2.5 gestation	2.78 gestation	52%	59.26%	≥ 8 on GAD‐7^9^	30.77	37.04
Werner 2016	54	30.87 (6.51)	29.60 (5.67)	9‐9.5 gestation		19.35 (13.79)	13.67 (10.11)	HAM‐A^10^	80.7	92.59

*Note*: ‘Control’ refers to randomised comparison groups only. Infant/fetal ages reported in weeks have been converted to months for interpretability (on the basis of 1 month = 4 weeks). * = informed by Lenze and Potts (2017). Anxiety measures as follows: 1 = the 6‐item State–Trait Anxiety Inventory (Brief STAI; Marteau & Bekker, 1992); 2 = Yale‐Brown Obsessive‐Compulsive Scale (YBOCS; Goodman et al., 1989); 3 = Depression Anxiety Stress Scales—anxiety scale (DASS; Lovibond & Lovibond, 1995); 4 = Strait Trait Anxiety Inventory—state scale (STAI‐S; Spielberger, 1970); 5 = Beck Anxiety Inventory (BAI; Beck & Steer, 1991); 6 = the 6‐item State‐Trait Anxiety Inventory—state scale (Brief STAI‐S; Berg et al., 1998; no interpretation of scores available); 7 = the Generalised Anxiety Disorder screening tool (GAD‐7; Spitzer et al., 2006); 8 = posttraumatic stress disorder or generalised anxiety disorder assessed via the Structured Clinical Interview for DSM‐IV‐R for Axis I disorders (SCID‐IV‐R; First et al., 1998); 9 = participants scoring ≥8 on the GAD‐7; 10 = Hamilton Anxiety Rating Scale (HAM‐A, Hamilton, 1959). Colour shading indicates anxiety severity level: orange—severe; yellow—moderate/’moderately severe’; green—mild/mild to moderate levels. Sources for interpretation of dimensional anxiety scores included relevant studies (e.g., Werner 2016 for HAM‐A), original work (e.g., Spitzer et al., 2006; GAD‐7) or the broader anxiety literature (e.g., Julian, 2011; BAI, STAI).

**TABLE 2:** Summary of findings table including details of participants, interventions, comparisons and outcomes, as well as effect sizes

Study author & year	Participants (N = total sample)	Intervention	Control	Parent anxiety outcome(s)	Infant/parent‐infant outcome(s)	Post‐intervention effect size (Hedges *g* calculated where possible)	
Country		N = total participants assigned to group		Measures and assessment timepoints		Parent anxiety	Infant/dyad
Burger 2020 Netherlands	Pregnant women screening positive for symptoms of depression (≥12 score on EDPS) and/or anxiety (≥42 score on STAI); once born, infants participated in the study up to 18 months postpartum (*N* = 282)	Prenatally initiated CBT: 10–14 × individual sessions (unspecified length) delivered from 20 weeks gestation to 3 months postpartum (6–10 sessions during pregnancy) (*N* = 140)	Care as usual (*N* = 142)	Brief STAI assessed at baseline, 24 and 36 weeks gestation and at 6 weeks and 3, 6, 12 and 18 months postpartum	CBCL—total problems, internalising, externalising scales; assessed at 18 months postpartum PBQ—between 6 and 18 months postpartum	Post‐intervention ratings, postpartum, for Brief STAI: 3 months: *g* = 0.21 6 months: *g* = 0.10 12 months: *g* = 0.03 18 months: *g* = 0.07	Post‐intervention ratings, 18 months postpartum for CBCL: Total problems: *g* = 0.17 Internalising: *g* = 0.22 Externalising: *g* = 0.08 PBQ: *g* = −0.10
Challacombe 2017 UK	Mothers diagnosed via SCID with OCD and an infant <6 months of age (*N* = 71)	Time intensive CBT (iCBT): Typically 4 × 3 h individual sessions, delivered in two weeks, with up to 3 × 1 h follow‐up sessions offered monthly (between 6 and 9 months postpartum) (*N* = 17)	Randomised treatment as usual (*N* = 17) Non‐randomised healthy controls (*N* = 37)	YBOCS and DASS assessed at baseline, and 6 and 12 months postpartum	(1) Ainsworth sensitivity scale; (2) Ainsworth cooperation‐interference scale; (3) Maternal warmth; (4) Maternal vocalisations during nappy change (%); (5) Over‐conscientiousness (%), and (6) Dyadic synchrony scale; all assessed at 6 and 12 months postpartum via videotaped interaction Attachment—assessed via Ainsworth SSP at 12 months postpartum	12‐month post‐intervention ratings for YBOCS: *g* = −0.91^†^ Pre/post DASS scores not reported in main paper	Not reported
Ericksen 2018 Australia	Mothers with an infant <12 months who had recently consulted with a health professional regarding their mental health (e.g., ‘symptoms of depression or anxiety’) (*N* = 31)	Community HUGS (CHUGS): 10 × 60–90 min therapeutic playgroup sessions targeting mother‐infant relationship over 10 weeks; 4–8 dyads in each group including interaction coaching, play, music, movement, and psychoeducation on CBT and parenting strategies (*N* = 16)	Wait‐list control, receiving care as usual (*N* = 15)	DASS assessed at baseline, post‐intervention (after session 10) and 6‐month follow‐up	PIPE scores PSI‐SF—parent‐child dysfunctional interaction scale, difficult child scale All assessed at baseline and post‐intervention (after session 10)	Not reported	Post‐intervention ratings (after 10 sessions): PIPE: *g* = 0.07 PSI difficult child: *g* = 0.29 PSI parent‐child dysfunctional interaction: *g* = 0.21
Goodman 2015 USA	Primiparous mothers with newborns, scoring >9 and <20 on the EPDS on two screens 1 week apart (*N* = 42)	Perinatal dyadic psychotherapy: 8 × 60 min individual sessions over 3 months; incorporates both standard and parent‐infant psychotherapy (*N* = 21)	Usual care (*N* = 21)	Any anxiety diagnosis assessed by SCID at post‐intervention and 3‐month follow‐up STAI‐state—assessed at baseline, post‐intervention and 3‐month follow‐up	PSI‐SF—total score CIB—maternal sensitivity, infant involvement and dyadic reciprocity assessed via videotaped interaction All assessed at post‐intervention and 3‐month follow‐up	Post‐intervention ratings:* STAI‐state: *g* = −0.42	Post‐intervention ratings:* Total PSI: *g* = −0.55 CIB Maternal sensitivity: *g* = 0.46 Infant involvement: *g* = 0.19 Dyadic reciprocity: *g* = 0.18
Holt 2021 Australia	Mothers with an infant <12 months meeting SCID diagnosis of current major or minor depressive disorder (*N* = 77)	CBT + HUGS: 12 × 90 min group CBT sessions (including 3 attended by partners) spread over 9 weeks, followed by 4 × 90 min therapeutic playgroup sessions including interaction coaching, ‘good enough’ parenting psychoeducation, play, and challenging infant‐centric cognitive distortions (*N* = 38)	CBT + control playgroup: CBT programme as per intervention group + 4 × 90 min nondirective group sessions with dyads and facilitator (*N* = 39)	BAI assessed at baseline, post‐CBT intervention, post HUGS intervention, and 6‐months follow‐up	ERA Factor I (FI) and items 19 and 22 assessed via videotaped interaction at baseline, post HUGS intervention, and 6‐months follow‐up PBQ, STSI/STST, ASQ:SE, PSI‐4 assessed at baseline, post CBT intervention, post HUGS intervention, and 6 months follow‐up	Post‐intervention ratings on the BAI: *g* = −0.10 6‐months follow‐up: *g* = 0.31	Post‐intervention measures:* PBQ: *g* = −0.26 ERA FI: *g* = 0.11 6‐months follow‐up: PBQ: *g* = −0.49^†^ ERA FI: *g* = 0.05^†^ ERA F1 effects calculated using adjusted means.
Lenze 2020 USA	Pregnant women between 12 and 30 weeks gestation scoring ≥10 on the EDS; once born, infants participated in the study up to 12 months postpartum (*N* = 42)	IPT‐Dyad: 9 × psychotherapy sessions (unspecified length) focused on the mother‐infant relationship, delivered during the prenatal period and followed up with up to 10 postpartum ‘maintenance’ sessions; including interaction coaching, exploration of maternal mental representations of the infant, and psychoeducation on attachment, developmental stages, and parenting (*N* = 21)	Enhanced treatment as usual: Regular contact; 15 nappies given per assessment; engagement with health services encouraged (*N* = 21)	Brief STAI‐S assessed at baseline, 37–39 weeks gestation, and 3, 6, 9 and 12 months postpartum	ITSEA—externalising, internalising, dysregulation and ‘competence’ scales. Assessed at 9 and 12 months postpartum IBQ‐VS—affect, control, surgency scales, and PSI—total score. Assessed at 6 and 12 months postpartum CIB—parent sensitivity, intrusiveness, and limit setting; child involvement; dyadic reciprocity, dyadic negative states. Assessed at 3, 6, 9 and 12 months postpartum	Not reported for parent or infant/dyad outcomes	
Milgrom 2015 Australia	Pregnant women <30 weeks gestation with a DSM‐IV diagnosis of minor or major depressive, once born, infants participated in the study up to 9 months postpartum (*N* = 54)	Beating the Blues Before Birth: 8 × 60 min individual sessions of pregnancy‐specific cognitive behavioural therapy, with one session including partners, over 8 weeks (*N* = 27)	Usual care (*N* = 27)	BAI assessed at baseline, 9 weeks post‐randomisation (post‐intervention), and at 9 months postpartum	ASQ:SE and IBQ‐R assessed at 9 months postpartum	Post‐intervention ratings of the BAI: 9‐weeks post‐randomisation: *g* = −0.90^†^ months postpartum: *g* = −0.64	Post‐intervention ratings at 9 months postpartum:* ASQ:SE self‐regulation: *g* = 0.83^†^ ASQ: SE communication: *g* = 0.82^†^ IBQ‐R falling reactivity/recovery: *g* = 1.08^†^ IBQ‐R negative affectivity: *g* = −0.85^†^ IBQ‐R high intensity pleasure: *g* = 0.83^†^
O’Higgins 2008 UK	Mothers of newborns scoring >12 on the EPDS (*N* = 96)	Infant massage class: 6 × 60 min group sessions, including training on various massage strokes and responsivity to infant cues (*N* = 31)	Randomised support group: Practical help on accessing helplines and welfare support (*N* = 31) Non‐randomised non‐depressed group (*N* = 34)	SSAI assessed at baseline, 19 weeks postpartum (post‐intervention) and 12 months postpartum	ICQ and Global Ratings for mother‐infant interaction (maternal sensitivity; infant performance in interaction; overall interaction)—all assessed at baseline, 19 weeks postpartum (post‐intervention) and 12 months postpartum	Not reported for parent or infant/dyad outcomes	
O’Mahen 2014 UK	Mothers who meet ICD‐10 criteria for major depressive disorder and who have a baby aged 0–12 months old (*N* = 83)	NetmumsHWD: 12 × individual online sessions, each designed to be completed in 1 week, supplemented with weekly 20–30 min phone call support and access to web resources (e.g. peer chat room and networking); five sessions focused on behavioural activation with the remainder addressing interpersonal issues, or parenting skills and infant behaviour (*N* = 41)	Treatment as usual (with access to NetmumsHWD web resources) (*N* = 42)	GAD‐7 assessed at baseline and 17 weeks post‐randomisation (post‐intervention)	PBQ assessed at baseline and 17 weeks post‐randomisation (post‐intervention)	Post‐intervention ratings: GAD‐7: *g* = −0.51^†^	Post‐intervention ratings: PBQ: *g* = −0.41
Stein 2018 UK	Mothers meeting diagnostic criteria for major depressive disorder and had been depressed for at least the previous 3 months or the first 3 months postpartum, along with their infants aged 4.5–9 months old (*N* = 144)	CBT + VFT; 11 × 90 min individual sessions of combined CBT + VFT (6 weekly and 5 fortnightly), followed by 2 post‐therapy boosters; VFT involves feedback on videotaped excerpts of dyadic interaction, plus coaching in parental responsivity, emotional scaffolding, sensitivity, and treating child as a psychological agent (*N* = 72)	CBT + PMR; 11 × 90 min individual sessions of combined CBT + PMR (6 weekly and 5 fortnightly), followed by 2 post‐therapy boosters; PMR involves tensing and relaxing major muscle groups combined with attention to sensations (*N* = 72)	GAD and PTSD assessed via SCID at baseline, and 12 and 24 months partum	CBCL externalising scale; AQS attachment security; child emotion regulation (Lab‐TAB), ECBQ effortful control, emotion discrimination (visual discrimination task) −all assessed and reported at two years postpartum Maternal following of child attention, responsivity, sensitivity, and warmth assessed and reported at baseline, 1 year and 2 years postpartum Maternal mind‐mindedness assessed and reported at baseline and 1 year postpartum	None reported for GAD and PTSD	Post‐intervention ratings at 2 years postpartum for primary measures:* CBCL externalising: *g* = −0.20 AQS attachment security: *g* = 0.09
Trevillion 2020 UK	Pregnant women at no further than 26 weeks gestation who met criteria for diagnostic depression or mixed anxiety and depressive disorder on the SCID (*N* = 53)	Usual care + guided self‐help: 8 × 30 min ∼weekly telephonic or face to face individual sessions, as well as a prior face‐to‐face initial session, and an additional telephone call at 6–8 weeks postpartum; involves working through a booklet including psychoeducation on prenatal depression, interpersonal issues, planning for parenthood and health and lifestyle (*N* = 26)	Usual care (*N* = 27)	GAD‐7 assessed at baseline, 14 weeks post‐randomisation, and 3 months postpartum	PBQ assessed and reported at 3 months postpartum	Adjusted odds ratio for GAD‐7. 14‐weeks post randomisation (post‐intervention but not postpartum): −0.48 3 months postpartum: −0.37	Statistics unavailable for calculating Hedges *g* but ‘effect size’ reported for post‐intervention ratings at 3 months postpartum: PBQ: −0.42
Werner 2016 USA	Pregnant women in their second or third trimester who scored ≥28 on the predictive index of postnatal depression (*N* = 54)	PREPP: 4 × individual sessions of unspecified length (3 in‐person visits, 1 telephone call) spanning the period between full term and 6 weeks postpartum; involving infant behavioural techniques (e.g., swaddling, increased carrying, daytime stimulation), as well as parent‐focused sessions on mindfulness, parental identity, and psychoeducation about the postpartum period (*N* = 27)	Enhanced treatment as usual; two in‐person meetings with a clinical psychologist (who discussed symptoms, offered referrals and provided printed support resources) and 1 telephone call from a research assistant (*N* = 27)	HAM‐A assessed at 34–38 weeks gestation (baseline), as well as 6, 10 and 16 weeks postpartum	Average infant fuss/cry behaviour assessed via parental diary; 4‐day average taken from 6 to 14 weeks postpartum	Post‐intervention, postpartum ratings for HAM‐A: 6 weeks: *g* = −0.29^†^ 10 weeks: *g* = −0.12 16 weeks: *g* = −0.23^†^	None reported for infant outcomes

*Note*: Hedges *g* has been calculated where means, standard deviations, and group sizes were reported at the timepoint for the measure of interest. Results based on dichotomous data have been presented as reported. A negative effect size corresponds to the control arm having a larger mean. For dimensional parent anxiety measures, as well as the CBCL, PBQ, PIPE, PSI, and IBQ‐R negative affectivity, higher scores indicate worse outcomes. For all other measures, higher scores indicate better outcomes. Only between group effects of outcomes applicable to the review are shown here. Where studies presented results from both observed and intention‐to‐treat (ITT) analyses, only results of the ITT analyses have been presented.

Abbreviations: ASQ:SE, Ages and Stages Questionnaires, Social Emotional (Squires et al., 2002); AQS, Attachment Q‐Sort (van IJzendoorn et al., 2004); BAI, Beck Anxiety Inventory (Beck & Steer, 1991); Brief STAI, six‐item State–Trait Anxiety Inventory (Marteau & Bekker, 1992); Brief STAI‐S, State Scale of the Brief STAI (Berg et al., 1998); CBCL, Child Behavioural Checklist (Rescorla, 2005); CBT, Cognitive Behavioural Therapy; EDS/EPDS, Edinburgh Postnatal (Depression) Scale (Cox et al., 1987); CIB, Coding Interactive Behaviour manual (Feldman, 1998); DASS, Depression Anxiety Stress Scales (Lovibond & Lovibond, 1995); ECBQ, Early Childhood Behaviour Questionnaire (Putnam et al., 2006); ERA, Parent Child Early Relational Assessment (Clark, 2015); GAD‐7, Generalised Anxiety Disorder Screener (Spitzer et al., 2006); HAM‐A, Hamilton Anxiety Rating Scale (Hamilton, 1959); IBQ‐R, Revised Infant Behaviour Questionnaire Short Form (Gartstein & Rothbart, 2003); IBQ‐VS, Infant Behaviour Questionnaire—Revised Very Short Form (Putnam et al., 2014); ICD‐10, International Classification of Diseases—version 10 (World Health Organization, 1990); ITSEA, Infant‐Toddler Social and Emotional Assessment (Carter et al., 1999); ITQ/ICQ, Bates Infant Temperament/Characteristics Questionnaire (Bates et al., 1979); Lab‐TAB, Laboratory Temperament Assessment Battery (Goldsmith & Rothbart, 1991); PBQ, Postpartum Bonding Questionnaire (Brockington et al., 2006); PIPE, Paediatric Infant Parent Exam (Fiese et al., 2001); PREPP—Practical Resources for Effective Postpartum Parenting (Werner et al., 2016); PMR, Progressive Muscle Relaxation (Carlson & Hoyle, 1993); PSI‐4, Parenting Stress Index (Abidin, 2012); PSI‐SF, Parenting Stress Index Short Form (Abidin, 1995); SSAI, Spielberger State Anxiety Inventory (Spielberger et al., 1970); SSP, Strange Situation Procedure (Ainsworth et al., 1978); STAI, State–Trait Anxiety Inventory (Spielberger et al., 1970); STSI, Short Temperament Scale for Infants (Sanson et al., 1987); STST, Short Temperament Scale for Toddlers (Sewell et al., 1988); VFT, Video Feedback Therapy (Juffer et al., 2008); YBOCS, [clinician‐rated] Yale‐Brown Obsessive‐Compulsive Scale (Goodman et al., 1989).

*Further fine‐grain non‐significant effect sizes from this study have been omitted from summary table due to volume of results.

^†^Statistically significant difference of at least *p* < 0.05.

**TABLE 3:** Summary of components of interventions with the potential to improve parent anxiety, infant development or parent‐infant relationship outcomes, split by study intervention focus

		Burger 2020	Challacombe 2017*	Milgrom 2015^†,^*	O’Mahen 2014*	Trevillion 2020	Ericksen 2018*	Goodman 2015	Holt 2021^†^	Lenze 2020	O’Higgins 2008	Stein 2018	Werner 2016^†,^*
		Interventions focused predominantly on the adult					Interventions focused predominantly on the infant or parent‐infant relationship						
1	Interaction coaching including support with how to read, understand and/or respond to infant cues						X	X	X	X	X	X	
2	Attachment‐based exploration of parent‐infant relationship						X			X		X	
3	Information on infant temperament and/or developmental stages						X	X		X			
4	Practical support in coping with infant behaviours such as colic, fussing, feeding and sleeping				X	X							X
5	Play therapy or sensory activities						X		X				
6	Treating infant as psychological agent											X	
7	Infant massage						X		X		X		
8	‘Good enough’ parenting principles				X				X				
9	Support with transition to parenthood, exploring changing roles and relationships, and balancing being a parent with being a person			X	X	X		X					
10	Psychotherapeutic approaches examining the parent's patterns of relating to others, for example, how their own childhood informs dyadic relationship, or exploration of maternal representations of parent and child							X	X	X			X
11	Cognitive behavioural strategies for anxiety, for example, exposure and responsive prevention exercises and cognitive‐restructuring; psychoeducation on perinatal anxiety may also be included	X	X		X		X						
12	Cognitive behavioural strategies for mood difficulties, for example, cognitive‐restructuring and problem‐solving; psychoeducation on perinatal depression may also be included	X		X		X							
13	Cognitive behavioural strategies for PTSD including exposure, imagery and rescripting work	X											
14	Behavioural activation	X			X								
15	Mindfulness training												X
16	Relaxation training			X					X				
17	Assistance with developing effective coping strategies for interpersonal problems, managing relationships and strengthening social networks			X	X	X				X			
18	Support with establishing a healthy lifestyle (e.g., sleep, self‐care)			X	X	X			X				
19	Resource‐based aid, for example, access to free baby care products									X			
20	Predominantly postnatal delivery		X		X		X	X	X	∼equal split	X	X	X
21	Predominantly prenatal delivery	X		X		X							
22	Group delivery						X		X		X		
23	Individual or dyadic delivery	X	X	X	X	X		X		X		X	X
24	Guided self‐help model (print or e‐resources with telephonic support)				X	X							
25	Intensive model (hours compressed to brief period)		X										
		Burger 2020	Challacombe 2017*	Milgrom 2015 ^†,^*	O’Mahen 2014*	Trevillion 2020	Ericksen 2018*	Goodman 2015	Holt 2021^†^	Lenze 2020	O’Higgins 2008	Stein 2018	Werner 2016^†,^*

*Note*: 1–10—components relating to the infant or parent‐infant relationship; 11–19—components relating to the adult; 20–25—components relating to the medium or format of delivery. A note on Stein et al. (2018) and Holt et al. (2021): as both these studies' intervention and active control groups were treated via a CBT programme prior to the main intervention of interest, only the main interventions are analysed and tabulated here (video feedback therapy and ‘HUGS’ therapeutic playgroup, respectively).

*Significant between group parent anxiety outcomes (*p* < 0.05).

^†^Significant between group infant/parent‐infant outcomes (*p* < 0.05).

### Study outcomes

Table 2 presents an overview of studies' participants, interventions, comparison groups, outcome measures, as well as effect sizes. Although practical time constraints and heterogeneity of outcome measures precluded formal meta‐analysis, Hedges *g* was calculated and reported where possible to aid interpretability. This was based on means, standard deviations, and group sizes available from the main trial article. Hedges' approach has the benefit of avoiding a slight overestimation bias compared to Cohen's *d* (Borenstein et al., 2009). Where studies derived their effect size from analyses of dichotomous data, odds ratios have been presented as in the original article. A guide to interpreting odds ratios in terms of effect sizes is given in the SM (Table S8). The below narrative synthesis relays study outcomes with a focus on magnitude of effect sizes, and statistical significance. Positive outcomes reflect an interaction between time and group (i.e., groups differences after and not before the intervention), unless otherwise specified.

#### Interventions examining between group improvements in parent anxiety outcomes

All 12 studies measured parent anxiety pre‐ and post‐intervention, with specific measures presented in Table 1 (10 out of 12 studies used self‐report measures). Details on the timepoints for each measure and control comparators for each intervention are provided in Table 2. Three studies reported post‐intervention changes in parent anxiety outcome that indicated medium to large effect sizes (Challacombe et al., 2017; Milgrom et al., 2015; O’Mahen et al., 2014). Challacombe et al. (2017), following a 2‐week CBT intervention at approximately 6 months postpartum, reported a large, significant between group effect size at 12 months postpartum, representing a reduction in OCD symptoms within the intervention group. Milgrom et al. (2015), following an 8‐week CBT intervention delivered in the prenatal period, also reported a large, significant effect at post‐intervention, representing a reduction in anxiety levels in the intervention group. However, this did not remain significant at 9 months postpartum (Milgrom et al., 2015). O’Mahen et al. (2014)—who examined a CBT‐based, behavioural activation and relapse‐prevention intervention—also reported a medium, significant between group effect post‐intervention, representing a reduction in anxiety for the intervention group.

Two studies found smaller or unidentifiable treatment effect sizes in relation to parent anxiety outcomes (Ericksen et al., 2018; Werner et al., 2016). Werner et al. (2016) examined an intervention using infant behavioural techniques as well as psychotherapy (psychoeducation, mindfulness, reflections on parental identity) that was conducted in the first 6 weeks postpartum. The authors found evidence that the intervention led to improved anxiety outcomes; significant reductions in anxiety symptoms were reported immediately post‐intervention (six weeks) and at a follow‐up assessment (16 weeks), albeit with a non‐significant reduction in the interim (10 weeks). These represented small effect sizes. Finally, Ericksen et al. (2018) investigated the effects of a therapeutic playgroup conducted in the infant's first year of life; significant between group differences were identified post‐intervention, representing a reduction in anxiety symptoms for the intervention group; however, this reduction in anxiety symptoms did not appear to be maintained, given analyses finding significant differences between post‐treatment and the 6‐month follow‐up for the intervention group (Ericksen et al., 2018). Effect sizes were not calculable for these results.

In addition, two studies indicated small to medium sized, directional improvements in parent anxiety such that anxiety reduced post‐intervention, though these were not found to reach significance when comparing groups. This included the guided self‐help intervention evaluated by Trevillion et al. (2020), and the dyadic psychotherapy intervention investigated by Goodman et al. (2015). One study, which evaluated a combined CBT and therapeutic playgroup intervention, found a small, directional improvement in anxiety for the index group post‐intervention, but this was not observed at the 6‐month follow‐up, and did not reach significance (Holt et al., 2021).

For the remaining four studies, it was not possible to calculate effect sizes for between group differences, nor were any significant between group differences identified. This included the combined CBT and video feedback therapy intervention investigated by Stein et al. (2018), the dyadic psychotherapy intervention studied by Lenze et al. (2020), and the infant massage intervention investigated by O’Higgins et al. (2008). The results of Burger et al. (2020) indicated that parental anxiety symptoms worsened during the intervention at 24 weeks gestation (such that anxiety scores were higher in the intervention group), but anxiety symptoms were not significantly different between pre‐intervention and follow‐up for the index or control group (see SM, Section 3).

#### Interventions examining between group improvements in infant/parent‐infant relationship outcomes

Ten studies measured parent‐infant relationship outcomes post‐intervention, using 24 different measures including both parent‐report measures and independent ratings of video‐taped interaction. Specific measures are presented in Table 1. Details on the timepoints for each measure and control comparators for each intervention are provided in Table 2. Multiple studies identified small to medium sized improvements in parent‐infant relationship outcomes. Firstly, Holt et al. (2021) used the Postpartum Bonding Questionnaire (PBQ; Brockington et al., 2006), a parent‐report measure capturing difficulties with parent‐infant bonding. Holt et al. (2021) also used the observer‐rated measure, the Parent Child Early Relational Assessment (ERA; Clark, 2015), specifically its first factor (‘Parental Positive Affective Involvement and Verbalisation’). The trial authors defined this as a measure of ‘maternal tone of voice, positive affect, mood, enjoyment in the interaction, amount and quality of visual contact and verbalisation with the child, social initiative with the child, structuring of the environment, mirroring, and consistency/predictability’ (Holt et al., 2021, p. 6). Following a two‐part intervention run over ∼13 weeks during the first year postpartum, Holt et al. (2021) reported small to medium effect sizes at 6‐month follow‐up that represented significant reductions in impaired bonding and significant improvements in positive parental involvement for the intervention group. Larger improvements in positive parental involvement were identified immediately post‐intervention in the intervention group compared to the control group, but between group differences were not significant until 6 months.

In addition to this, both Trevillion et al. (2020) and O’Mahen et al. (2014) observed a medium sized, directional improvement on the PBQ (Brockington et al., 2006), while Burger et al. (2020) observed a similar pattern, though with a smaller effect size. Goodman et al. (2015) found small to medium treatment effects on several dyadic behaviours assessed using the Coding Interactive Behaviour manual (dyadic reciprocity, infant involvement, maternal sensitivity; Feldman, 1998) and the Parenting Stress Index (PSI; Abidin, 1995). Stein et al. (2018) found small treatment effects indicative of increased attachment security, measured by the Attachment *Q* Sort (AQS; van IJzendoorn et al., 2004). None of these effects were statistically significant.

Eight studies measured infant socio‐emotional temperament or development outcomes post‐intervention (all eight involved parent‐report measures, alongside one use of response to experimental stimuli; Stein et al., 2018). Several studies identified improvements in infant socio‐emotional functioning. Stein et al. (2018) found small treatment effects indicative of reduced child externalising behaviour, measured by the Child Behavioural Checklist (CBCL; Rescorla, 2005), though these were not significant. Milgrom et al. (2015) used two parent‐report measures: the Social‐Emotional Ages and Stages Questionnaires (ASQ:SE; Squires et al., 2002), and the Revised Infant Behaviour Questionnaire Short Form (IBQ‐R; Gartstein & Rothbart, 2003). Following an 8‐week intervention conducted during the prenatal period, Milgrom et al. (2015) reported large treatment effects at 9 months postpartum that represented significant differences in measures of infant self‐regulatory and communicative behaviours. Those in the intervention group scored higher on three scales probing self‐regulation (see Table 2). However, these measures were only assessed at 9 months postpartum, precluding any analyses of change over time.

Werner et al. (2016) also examined between group differences in infant fussing and crying behaviour, using the Baby's Day Diary (Barr et al., 1988), a parent‐report measure. Fuss and cry behaviour is closely related to the temperament construct of soothability, that is, the extent to which reductions in infant fuss and cry behaviour occur in the context of caregiver soothing techniques (Gartstein & Rothbart, 2003). Following an intervention delivered over 6 weeks postpartum, infants in the intervention group exhibited significantly fewer episodes of fuss/cry behaviour based on a 4‐day average collected post‐intervention. Effect sizes were not calculable.

With respect to infant or dyadic outcomes, effect sizes indicating between group differences were not calculable for Lenze et al. (2020), O’Higgins et al. (2008), or Challacombe et al. (2017), and none reported statistically significant improvements. The results of Ericksen et al. (2018) indicated adverse treatment side‐effects for parent‐infant relationship outcomes (see SM, Section 3), though these analyses were underpowered. All infant and dyadic outcome measures for each study are shown in Table 2.

### Intervention components analysis

To probe the study findings further and examine the mechanisms of improved treatment outcome, an analysis of intervention components was conducted from which two broad groupings emerged. One grouping, ‘interventions predominantly focused on the adult’ included interventions with more adult‐focused than infant/dyad‐focused components. The second grouping, ‘interventions predominantly focused on the infant or parent‐infant relationship,’ included interventions with more infant and dyad‐focused than adult‐focused components.

During this analysis, 10 infant or dyad‐focused components were identified. These included: interaction coaching including support with how to read, understand and/or respond to infant cues; attachment‐based exploration of the parent‐infant relationship; information on infant temperament and/or developmental stages; practical support in coping with infant behaviours such as colic, fussing, feeding and sleeping; play therapy or sensory activities; treating the infant as a psychological agent; infant massage; ‘good enough’ parenting principles; support with transition to parenthood, and psychotherapeutic approaches examining the parent's patterns of relating to others, including exploration of maternal representations of the child, and examination of how the parent's own childhood informs the dyadic relationship.

Nine adult‐focused intervention components were also identified. These were: cognitive behavioural strategies for mood difficulties, anxiety and PTSD; behavioural activation; mindfulness training; relaxation training; assistance with developing effective coping strategies for interpersonal problems and managing relationships; support with establishing a healthy lifestyle, and resource‐based aid (e.g., access to free baby‐care products). The intervention components matrix also included components related to the format of delivery (e.g., prenatal *v.* postnatal, individual *v.* group sessions).

All the intervention components and significant results were identified from studies and mapped onto the matrix. From this we were able to identify symmetrical effects and asymmetrical effects, as described in the Methods. The matrix also allowed us to consider whether there were common components among interventions that demonstrated significant improvements in outcomes of interest. The matrix is presented in Table 3.

#### How adult‐focused interventions affected adults

Five studies investigated mostly adult‐focused interventions (Burger et al., 2020; Challacombe et al., 2017; Milgrom et al., 2015; O’Mahen et al., 2014; Trevillion et al., 2020). All five measured changes in parental anxiety. Of these, three led to significantly improved parent anxiety scores, with medium to large effect sizes (Challacombe et al., 2017; Milgrom et al., 2015; O’Mahen et al., 2014). Trevillion et al. (2020) also demonstrated non‐significant, small directional improvement in parent anxiety. As discussed earlier, Burger et al. (2020) did not demonstrate such improvement and found significant adverse treatment effects on parent anxiety during the intervention.

#### How adult‐focused interventions affected infants or the parent‐infant relationship

Of the five studies investigating mostly adult‐focused interventions (Burger et al., 2020; Challacombe et al., 2017; Milgrom et al., 2015; O’Mahen et al., 2014; Trevillion et al., 2020), two measured levels of infant socio‐emotional functioning. One of these (Milgrom et al., 2015) found higher ratings of infant social and emotional competencies, as well as lower negative affect and greater high intensity pleasure, in infants in the intervention group compared to the control condition; these represented large effect sizes. Four of the five adult‐focused interventions also included measures of the quality of the parent‐infant relationship. Of these, two interventions demonstrated directional non‐significant improvements in parent‐infant bonding (O’Mahen et al., 2014; Trevillion et al., 2020).

No improvements in either infant socio‐emotional development or parent‐infant relationship outcomes were demonstrated by the other adult‐focused interventions (Burger et al., 2020; Challacombe et al., 2017).

#### How infant‐focused interventions affected infants or the parent‐infant relationship

Seven studies investigated mostly infant or dyad‐focused interventions (Ericksen et al., 2018; Goodman et al., 2015; Holt et al., 2021; Lenze et al., 2020; O’Higgins et al., 2008; Stein et al., 2018; Werner et al., 2016). Of these, six measured the quality of the parent‐infant relationship and six measured levels of infant socio‐emotional functioning. One intervention led to significant improvements in the parent‐infant relationship, with small effect sizes; Holt et al. (2021) found statistically significant improvements in positive parental involvement and parent‐infant bonding in the intervention group compared to the control condition. Non‐significant directional improvements in the parent‐infant relationship were also found by Goodman et al. (2015).

Six of the seven infant‐focused interventions also measured levels of infant socio‐emotional functioning. Of these, one intervention led to significant improvements in infant socio‐emotional functioning; Werner et al. (2016) found significantly lower rates of infant fuss/cry behaviour in the intervention group compared to the control condition. Non‐significant directional improvements in infant socio‐emotional competencies were found by Stein et al. (2018).

No improvements in either infant socio‐emotional development or parent‐infant relationship outcomes were demonstrated by the other infant‐focused interventions (Ericksen et al., 2018; Lenze et al., 2020; O’Higgins et al., 2008). As discussed above, Ericksen et al. (2018) found adverse treatment effects on the parent‐infant relationship, but these were non‐significant and likely the result of underpowered analyses.

#### How infant or dyad‐focused interventions affected adults

Of the seven studies investigating mostly infant or dyad‐focused interventions (Ericksen et al., 2018; Goodman et al., 2015; Holt et al., 2021; Lenze et al., 2020; O’Higgins et al., 2008; Stein et al., 2018; Werner et al., 2016), all measured changes in parental anxiety. Of these, two studies found evidence that post‐intervention ratings of parent anxiety scores were significantly lower in the intervention group compared to the control condition (Ericksen et al., 2018; Werner et al., 2016). These represented small effect sizes within potentially underpowered studies. Similarly, Goodman et al. (2015), an infant‐focused intervention, demonstrated non‐significant directional improvement in parent anxiety. When comparing groups, parent anxiety scores also appeared to improve post‐intervention in Holt et al. (2021)—but only temporarily. No such improvements in anxiety were identified in the remaining infant‐focused interventions (Lenze et al., 2020; O’Higgins et al., 2008; Stein et al., 2018).

### Components common to successful interventions

The intervention components matrix allowed conclusions to be drawn regarding the extent to which interventions focusing on one partner would lead to improved outcomes in the other. Additionally, though the overall number of studies in the review was small, the components matrix allowed patterns to be observed among ‘successful’ interventions (i.e., those demonstrating significant improvements). As shown by Table 3, interventions that demonstrated significant (medium sized) improvements in parent anxiety shared a focus on cognitive behavioural strategies for mood or anxiety (Challacombe et al., 2017; Milgrom et al., 2015; O’Mahen et al., 2014). In addition, interventions demonstrating significant (small) improvements in infant and parent‐infant relationship outcomes shared a focus on the exploration of distorted maternal representations (Holt et al., 2021; Werner et al., 2016). A component‐by‐component breakdown of adult‐focused and infant/dyad‐focused interventions is included in the SM (Sections DISCUSSION, AUTHOR CONTRIBUTIONS).

## DISCUSSION

The present review examined the efficacy of a range of perinatal interventions with regard to their effect on parent anxiety outcomes, parent‐infant relationship outcomes, and socio‐emotional development or temperament outcomes. Twelve studies were systematically retrieved and included, with no restrictions on whether parent anxiety outcomes were operationalised categorically or dimensionally. The analysis comprised of narrative reporting on the original studies, as well as identifying common components among successful interventions, that is, those that led to significant improvements in outcomes of interest. The potential for predominantly adult‐focused interventions to improve infant or dyad‐related outcomes (and for predominantly infant/dyad‐focused interventions to improve adult outcomes) was also explored. This analysis was conducted in an effort to focus on mechanisms of treatment outcomes that may be informative for trialling and translating future interventions. Importantly, statistical power was limited for the majority of studies included in this review; the evidence amassed must therefore be treated as preliminary and interpreted with caution.

Firstly, this review evaluated whether parent‐focused perinatal interventions led to improvements in parent anxiety, and what commonalities were present among successful interventions. Of five interventions that were mostly adult‐focused, three were found to significantly improve parent anxiety symptoms (Challacombe et al., 2017; Milgrom et al., 2015; O’Mahen et al., 2014). These three interventions all incorporated components from cognitive behavioural therapy (e.g., cognitive‐restructuring) and generated medium to large effects; all interventions were delivered postnatally, except one (Milgrom et al., 2015). The prenatal, guided self‐help intervention investigated by Trevillion et al. (2020) also demonstrated directional improvement in parent anxiety. Though these results were not significant, they were nonetheless consistent with the overall pattern of favourable results for CBT. By contrast, the prenatal CBT intervention investigated by Burger et al. (2020) found evidence that diverged from this. Prenatal CBT was related to a medium sized, significant increase in parent anxiety during pregnancy, as well as a (non‐significant) elevation in anxiety post‐intervention, after 3 months post‐partum. The increase in anxiety during pregnancy was associated with adverse birth outcomes among infants of anxious parents in the intervention group, theorised by Burger et al. (2020) to be a consequence of CBT exposure exercises and the increased physiological stress likely triggered by them (see SM, Section 3).

Given links between prenatal physiological hyperarousal and adverse birth outcomes, researchers have questioned whether exposure‐based cognitive behavioural therapies are advisable during pregnancy; however, researchers have also argued that the risks of exposure‐based CBT approaches are outweighed by the relatively greater risk of untreated anxiety presentations—and associated physiological stressors—during pregnancy (Arch et al., 2012). In addition, reviews of clinical treatment for perinatal anxiety, which include numerous patients receiving care in the prenatal period, have found significant, medium to large (unpooled) effects of CBT programmes on parental anxiety symptoms (Loughnan et al., 2018), as well as small between group effects and large within group effects of pooled controlled and uncontrolled CBT trials (Maguire et al., 2018). This would appear to conflict with the findings from the amply powered study of prenatal provision investigated by Burger et al. (2020), who found that CBT did not improve reduce perinatal anxiety symptoms (and that CBT was associated with other side‐effects for infants of anxious mothers). However, it is important to note that the above reviews represent mostly small pilot studies, as well as a mixture of postnatal and prenatal patients (Loughnan et al., 2018; Maguire et al., 2018). In addition, reviews of psychotherapeutic interventions should be interpreted cautiously given systemic issues in the field of clinical psychological research. Studies with unfavourable treatment outcomes are less likely to be published (publication bias), and studies in psychotherapy research tend to be biased towards the main authors' psychotherapeutic allegiance (allegiance bias) (Hengartner, 2018). Overall, however, the results from this review and the wider literature suggest that CBT for perinatal anxiety appears to be an effective treatment option for reducing parent anxiety.

Secondly, we looked at whether infant or dyad‐focused perinatal interventions led to improved outcomes for the parent's anxiety and—if so—what successful interventions had in common. Of seven interventions focused on the infant or dyad, two were found to significantly improve parent anxiety outcomes (Ericksen et al., 2018; Werner et al., 2016). These two interventions shared no components (apart from a predominantly postnatal delivery format). In addition, Werner et al. (2016) was judged to be at high risk of bias due to missing outcome data and the possibility of selective reporting (see Figure 2), limiting interpretation of its effects.

Ericksen et al. (2018) evaluated a predominantly infant‐focused intervention. Interestingly, this did not lead to significant improvements in infant outcomes, but led to reduced anxiety scores among parents. It is possible that equipping parents with a greater understanding of dyadic interaction and infants' regulatory needs increases belief in parenting capacities, in turn reducing anxiety levels. This is suggested by research showing that negative thoughts about parenting efficacy are associated with greater perinatal anxiety and depression (O’Mahen et al., 2012; Sockol et al., 2014). However, the reduction in anxiety symptoms found by Ericksen et al. (2018) did not appear to be maintained, given analyses finding significant differences between post‐treatment and the 6‐month follow‐up for the intervention group. In addition, though other dyad‐focused interventions led to directional, non‐significant improvements in parent anxiety outcomes when comparing intervention and control groups (e.g., Goodman et al., 2015; see also Holt et al., 2021), it was not possible to identify this in trials of other infant‐focused interventions (Lenze et al., 2020; O’Higgins et al., 2008; Stein et al., 2018).

Next, we evaluated whether infant or dyad‐focused perinatal interventions led to improved outcomes for the infant/dyad, and what successful interventions had in common. Of the seven interventions focused on the infant/dyad, two interventions were found to significantly improve infant or parent‐infant outcomes (Holt et al., 2021; Werner et al., 2016). These generated small to medium effects, and shared a focus on distorted maternal internal representations of the child or parent. Non‐significant directional improvements in infant socio‐emotional competency and the dyadic relationship were further demonstrated by interventions looking at related approaches, including sensitising mothers to their infants' ‘uniqueness’, and treating the infant as a psychological agent (Goodman et al., 2015; Stein et al., 2018). Improvements in dyadic or infant outcomes were not demonstrated in two small, underpowered pilot studies (Ericksen et al., 2018; Lenze et al., 2020), nor a study of an infant massage intervention (O’Higgins et al., 2008).

There is also some evidence that interventions focused on distorted mental representations (Ahlfs‐Dunn et al., 2021; Guyon‐Harris et al., 2021) can be effective in preventing socio‐emotional difficulties arising from overly involved dyadic relations (Holt et al., 2021; Werner et al., 2016), although these studies were judged to be of high risk of bias due to the possibility of selective reporting (see Figure 2).

Finally, we looked at whether *adult* focused perinatal interventions led to improved outcomes for the infant or dyad, and what any potentially successful interventions had in common. Of five adult‐focused interventions, one intervention was found to significantly improve infant socio‐emotional development outcomes, with large effects (Milgrom et al., 2015). The three adult‐focused, CBT‐based interventions also demonstrated small to medium directional improvements in parent‐infant bonding (Burger et al., 2020; O’Mahen et al., 2014; Trevillion et al., 2020), though these did not reach significance. No such improvements were demonstrated by the remaining intervention (Challacombe et al., 2017). These results are consistent with evidence suggesting that perinatal interventions focusing only on parental mood are insufficient for establishing improvements in child/dyadic outcomes (Stein et al., 2014): only one of the five adult‐focused interventions led to a statistically significant improvement in child/dyadic outcomes, in line with evidence that suggests treatment may need to target both parent *and* child/dyadic factors (Stein et al., 2014). This would be coherent with theoretical perspectives suggesting that the transactional relations between parent and child are central to the development of typical emotion regulation (Gouze et al., 2017; Smith, 2022; Yirmiya et al., 2021). It is also worth noting that most of these studies were not powered to detect infant or dyadic outcomes (Milgrom et al., 2015; O’Mahen et al., 2014; Trevillion et al., 2020).

It is important to acknowledge that improvements in infant outcomes may have been related to treatment affecting parental depression as well as—or instead of—anxiety. Co‐occurring depression and anxiety are highly prevalent in the perinatal period (Falah‐Hassani et al., 2017), and this is reflected in most participants' high baseline anxiety scores (Table 1) alongside the presence of depression symptoms (Table 2). Parental depression is also known to impact on parent‐infant interaction and infant development (Gueron‐Sela et al., 2018; Stein et al., 2014). Interventions evaluated in the present review may have ameliorated depressive symptoms. This is perhaps through helping parents to reduce negative appraisals regarding their infant or their own parenting abilities (Dix & Meunier, 2009; Dix & Moed, 2019). This in turn may explain improved parent‐infant interaction outcomes. Alternatively, interventions could have led to a reduction in depressive symptoms, thus facilitating a reduction in parental anxiety symptoms. This may have been due to reductions in shared maintenance processes for depression and anxiety, such as avoidance (Grant et al., 2013). This may consequently have reduced aspects of intrusiveness or overstimulation in the parent‐infant relationship, explaining improved parent‐infant interaction and infant outcomes.

### General conclusions

This review examined the efficacy of perinatal interventions with respect to parent anxiety outcomes, parent‐infant relationship outcomes, and infant socio‐emotional outcomes. There were three main conclusions. Firstly, interventions incorporating cognitive behavioural strategies have the potential to demonstrate improvements in parent anxiety outcomes during the perinatal period. This finding extends our understanding of the efficacy of CBT for anxiety by suggesting its application in the perinatal period as in the general population (Cuijpers et al., 2016).

Secondly, interventions addressing distorted maternal representations, and potentially emphasising the infant's uniqueness/individual agency, may facilitate improvements in the parent‐infant relationship or infant socio‐emotional functioning.

Thirdly, there is limited evidence to suggest that adult‐focused interventions demonstrate improvements in infant or dyadic outcomes (and infant/dyadic‐focused interventions improve adult outcomes). Studies showing ‘asymmetrical’ intervention effects were constrained by low statistical power, raising questions over their validity (Ericksen et al., 2018; Milgrom et al., 2015; Werner et al., 2016). In addition, transactional models of intervention have highlighted the importance of integrating both parents and children into treatment programmes, on the basis that socio‐emotional difficulties in one partner tend to exacerbate difficulties in the other (Sameroff & Fiese, 1990).

### Implications for practice and future trials

The present review has several implications for clinical practice. Evidence from included studies indicates that interventions for perinatal anxiety may benefit from being informed by CBT strategies, such as cognitive‐restructuring. Efforts to minimise difficulties in infant socio‐emotional development or the parent‐infant relationship in the context of perinatal anxiety may also benefit from addressing distorted maternal internal representations, and highlighting the infant as a unique, individual agent. They could also focus on interaction dynamics, targeting parental over‐reactivity to minor physiological stress events (Smith et al., 2021) and arousal‐triggering parental vocal behaviour (Smith, 2022; Smith et al., 2022). These practices could be incorporated in therapeutic approaches that focus on minimising distress within the parent‐infant relationship, such as parent‐infant psychotherapy or parent‐infant video feedback therapy.

### Implications for future intervention trials

The results of this review have implications for the design of future trials evaluating interventions for perinatal anxiety and infant outcomes. Firstly, trials may benefit from a focus on anxiety distinct from depression. Trials included in the present review often recruited from populations at risk of depression and anxiety, or depression only. This is representative of the traditional dominance of research on perinatal depression compared with other perinatal mental illnesses (Howard et al., 2014). While anxiety and depression often co‐occur and share diagnostic features (Falah‐Hassani et al., 2017; Grisanzio et al., 2018), the two conditions exert substantively different effects on the parent‐infant relationship in the first year of life (Feldman, 2007; Feldman et al., 2009). Anxious parents also have different biobehavioural patterns of relating to their infants compared with non‐anxious or depressed parents (Amole et al., 2017; Granat et al., 2017; Smith et al., 2021). As such, future trials examining interventions specialised for perinatal anxiety may prove to have more substantial benefits for the infants of anxious parents. An example of this approach is already underway (Wilkinson et al., 2016).

Secondly, trials focusing on the mechanisms by which perinatal anxiety leads to atypical socio‐emotional function in infants are needed. From multifactorial, complex interventions, it is not clear which of these components maps to specific outcomes. Dismantling studies, which experimentally manipulate specific components of interventions, may elucidate which aspect of a perinatal intervention includes the active mechanism of change (Gaudiano, 2008; Papa & Follette, 2015).

Finally, this review has highlighted a need for more adequately powered analyses, which may aid more mechanistic analyses of moderation and mediation. This is in contrast to the pilot trials included in this review, which were not powered to detect small to medium effects (though in some instances power calculations were not stated at all; Challacombe et al., 2017; Werner et al., 2016). Where trials are conducted in the future, these should be accompanied by pre‐specified and detailed analyses plans, allowing for an informed risk of bias assessment. Future trials may also benefit from including fathers and non‐binary parents, alongside mothers, to augment generalisation.

### Strengths and limitations

This review is characterised by several strengths. The search strategy was comprehensive, including five electronic search databases from a range of disciplines, and multiple manual search procedures. Given that perinatal anxiety is an under‐researched area compared to other perinatal disorders, the broadness of search terms allowed us to retrieve records that included but did not foreground parent anxiety outcomes. Study screening, data extraction, and risk of bias assessments were conducted according to best practice in systematic reviewing; this included independent coding from two reviewers during title and abstract screening, team verification of included studies, and a discrepancy check on risk of bias assessments.

The review was also subject to several limitations. Firstly, time constraints prevented the searching of grey literature. This may have introduced a degree of publication bias and precluded the inclusion of studies with more diverse samples. Due to the heterogeneity of study outcomes, our analytical strategy was also limited to a pragmatic, narrative synthesis, which introduced greater subjectivity than quantitative approaches such as meta‐analyses. Our approach of grouping studies into ‘infant/dyad‐focused’ or ‘adult‐focused’ interventions was also reductive, and did not allow for conclusions to be drawn about interventions that targeted both parent *and* infant equally (nor for individualised CBT interventions that could have included therapeutic goals focused on parenting). These conceptual and methodological issues could inform future meta‐analyses evaluating interventions affecting perinatal anxiety and infant socio‐emotional development. Lastly, bias assessments were conducted by researchers at the pre‐doctoral level. Recent controversies surrounding inaccurate bias assessments have highlighted the need for assessors with expertise in forensic numerical data analysis to be involved in quality assessment procedures for reviews of therapeutics (Brown, 2021; Davey, 2021; Meyerowitz‐Katz, 2021).

## AUTHOR CONTRIBUTIONS

**Celia G. Smith**: Conceptualization, Data curation, Formal analysis, Funding acquisition, Investigation, Methodology, Project administration, Resources, Visualization, Writing – original draft, Writing – review & editing. **Emily J. H. Jones**: Conceptualization, Funding acquisition, Supervision, Writing – review & editing. **Sam V. Wass**: Conceptualization, Funding acquisition, Resources, Supervision, Writing – review & editing. **Dean Jacobs**: Formal analysis, Investigation, Validation, Writing – review & editing. **Cassie Fitzpatrick**: Formal analysis, Investigation, Validation, Writing – review & editing. **Tony Charman**: Conceptualization, Funding acquisition, Supervision, Writing – review & editing.

## CONFLICTS OF INTEREST

Emily Jones is a Joint Editor for JCPP *Advances*. The remaining authors have declared that they have no competing or potential conflicts of interest.

## ETHICAL CONSIDERATIONS

This study was a systematic review of literature related to the review topic. As it did not involve participation of human subjects or the use of secondary data, ethical approval was not required or sought.

## Supporting information

Supporting Information S1

## Data Availability

The analysis of this review was based on existing data, which are openly available at locations cited in the reference section.
