# Supplementary material for: The effect of perinatal interventions on parent anxiety, infant socio‐emotional development and parent‐infant relationship outcomes: A systematic review
Source: JCPP Adv. 2022 Nov 19;2(4):e12116. doi: 10.1002/jcv2.12116 (PMC10242933; doi:10.1002/jcv2.12116)
Supplement: Supplementary file 1 — Supporting Information S1 [file JCV2-2-e12116-s001.docx]

# Supporting Information for: The effect of perinatal interventions on parent anxiety, infant socio-emotional development and parent-infant relationship outcomes: a systematic review

**Table of Contents**

[Table S1 Electronic database search terms optimised for Medline. 55](#_Toc85371803)

[Table S2 Electronic database search terms optimised for Embase. 56](#_Toc85371804)

[Table S3 Electronic database search terms optimised for APA PsychINFO. 57](#_Toc85371805)

[Table S4 Electronic database search terms optimised for MIDIRS. 58](#_Toc85371806)

[Table S5 Electronic database search terms optimised for Cochrane. 59](#_Toc85371807)

[Table S6 Reasons for exclusion for articles assessed at full-text. 60](#_Toc85371808)

[Table S7 Reasons for inclusion for articles assessed at full-text 84](#_Toc85371809)

[1 Intervening for perinatal anxiety v. intervening for broad risk or transdiagnostic symptoms 88](#_Toc85371810)

[2 Risk of bias assessments 88](#_Toc85371811)

[Table S8 An approximate guide to interpreting the strengths of associations represented by Hedges g/Cohen’s d, as well as odds ratios. 89](#_Toc85371812)

[3 Component-by-component breakdown of adult-focused interventions 89](#_Toc85371813)

[4 Component-by-component breakdown of infant or dyad-focused interventions 90](#_Toc85371814)

| **#** | **Terms** |
| --- | --- |
| 1 | (parent* adj5 (mental* ill* or mental* disorder* or mental health or mood disorder* or affective disorder or anxi* or depress* or OCD or obsessive compulsive disorder or PTSD or post traumatic stress disorder or trauma)).mp. |
| 2 | exp PARENTS/ and (exp Mental Disorders/ or exp Mental Health/ or exp Mood Disorders/) |
| 3 | 1 or 2 |
| 4 | (intervent* or prevent* or therap* or train* or program* or treatment).mp. |
| 5 | Exp Psychotherapy/ or exp Health Services/ |
| 6 | 4 or 5 |
| 7 | exp Parent-Child Relations/ or exp Child Rearing/ or exp Infant Behavior/ or exp Infant development/ |
| 8 | ((mother* or maternal) adj5 (infant* or baby or child*) adj5 (interact* or relations* or bond* or develop*)).mp. |
| 9 | 7 or 8 |
| 10 | 3 and 6 and 9 |
| 11 | randomized.mp. |
| 12 | placebo.mp. |
| 13 | randomly.mp. |
| 14 | trial.mp. |
| 15 | 11 or 12 or 13 or 14 |
| 16 | 10 and 15 |

## Table S1 Electronic database search terms optimised for Medline.

| **#** | **Terms** |
| --- | --- |
| 1 | (parent* adj5 (mental disease* or mental health or mood disorder* or anxi* or depress* or obsessive compulsive disorder or posttraumatic stress disorder)).mp. |
| 2 | exp PARENT/ and (exp Mental Disorders/ or exp Mental Health/ or exp Mood Disorder/) |
| 3 | 1 or 2 |
| 4 | (intervent* or prevent* or therapy* or train* or health program or treatment).mp. |
| 5 | exp Psychotherapy/ or exp Health Program/ |
| 6 | 4 or 5 |
| 7 | exp child parent relation/ or exp child rearing/ or exp child behavior/ or exp child development/ |
| 8 | ((mother* or maternal) adj5 (infant* or baby or child*) adj5 (interact* or relations* or bond* or develop*)).mp. |
| 9 | 7 or 8 |
| 10 | 3 and 6 and 9 |
| 11 | randomized controlled trial.mp. |
| 12 | placebo.mp. |
| 13 | 11 or 12 |
| 14 | 10 and 13 |

## Table S2 Electronic database search terms optimised for Embase.

| **#** | **Terms** |
| --- | --- |
| 1 | (parent* adj5 (mental disorder* or mental health or affective disorder* or anxi* or postpartum depression or major depression or obsessive compulsive disorder or posttraumatic stress disorder or birth trauma)).mp. |
| 2 | exp PARENTS/ and (exp Mental Disorders/ or exp Mental Health/ or exp Affective Disorders/) |
| 3 | 1 or 2 |
| 4 | (intervent* or prevent* or psychotherap* or train* or mental health programs or treatment).mp |
| 5 | exp Psychotherapy/ or exp Intervention/ |
| 6 | 4 or 5 |
| 7 | exp Parent Child Relations/ or exp Childrearing Practices/ or exp Infant Temperament/ or exp Infant Development/ |
| 8 | ((mothers or maternal) adj5 (infant* or baby or child*) adj5 (interact* or relations* or bond* or develop*)).mp. |
| 9 | 7 or 8 |
| 10 | 3 and 6 and 9 |
| 11 | Randomized controlled trials.mp. |
| 12 | placebo.mp. |
| 13 | Control Groups.mp. |
| 14 | clinical trials.mp. |
| 15 | 11 or 12 or 13 or 14 |
| 16 | 10 and 15 |

## Table S3 Electronic database search terms optimised for APA PsychINFO.

| **#** | **Terms** |
| --- | --- |
| 1 | (parent* adj5 (mental* ill* or mental* disorder* or mental health or mood disorder* or affective disorder or anxi* or depress* or OCD or obsessive compulsive disorder or PTSD or post traumatic stress disorder or trauma)).mp. |
| 2 | (intervent* or prevent* or therap* or train* or program* or treatment).mp. |
| 3 | ((mother* or maternal or parent or parental) adj5 (infant* or baby or child*) adj5 (interact* or relations* or bond* or develop*)).mp. |
| 4 | randomized.mp. |
| 5 | placebo.mp. |
| 6 | randomly.mp. |
| 7 | controlled trial.mp. |
| 8 | 4 or 5 or 6 or 7 |
| 9 | 1 and 2 and 3 and 8 |

## Table S4 Electronic database search terms optimised for MIDIRS.

| 1 | parent* adj5 (mental* ill* or mental* disorder* or mental health or mental disease* or mood disorder* or affective disorder* or anxi* or depress* or OCD or obsessive compulsive disorder or PTSD or post traumatic stress disorder or trauma) | Limits |
| --- | --- | --- |
| 2 | Mesh descriptor: [Parenting] in all MeSH products | MeSH |
| 3 | Mesh descriptor: [Mental Disorders] explode all trees | MeSH |
| 4 | Mesh descriptor: [Mental Health] explode all trees | MeSH |
| 5 | Mesh descriptor: [Mood Disorders] explode all trees | MeSH |
| 6 | #2 AND (#3 OR #4 or #5) | Limits |
| 7 | #1 or #6 | Limits |
| 8 | intervent* or prevent* or therap* or train* or program* or treatment | Limits |
| 9 | Mesh descriptor: [Psychotherapy] explode all trees | MeSH |
| 10 | Mesh descriptor: [National Health Programs] explode all trees | MeSH |
| 11 | Mesh descriptor: [Health Services] explode all trees | MeSH |
| 12 | #9 or #10 or #11 | Limits |
| 13 | #8 or #12 | Limits |
| 14 | MeSH descriptor: [Parent-Child Relations] explode all trees | MeSH |
| 15 | MeSH descriptor: [Child Rearing] explode all trees | MeSH |
| 16 | MeSH descriptor: [Child Behavior] explode all trees | MeSH |
| 17 | MeSH descriptor: [Child Development] explode all trees | MeSH |
| 18 | #14 or #15 or #16 or #17 | Limits |
| 19 | (mother* or maternal) adj5 (infant* or baby or child*) adj5 (interact* or relations* or bond* or develop*) | Limits |
| 20 | #18 or #19 | Limits |
| 21 | #7 and #13 and #18 | Limits |
| 22 | MeSH descriptor: [Randomized Controlled Trials] explode all trees | MeSH |
| 23 | MeSH descriptor: [Random Allocation] explode all trees | MeSH |
| 24 | #22 OR #23 | Limits |
| 25 | #21 AND #24 | Limits |

## Table S5 Electronic database search terms optimised for Cochrane.

## Table S6 Reasons for exclusion for articles assessed at full-text. Where ‘duplicate’ is given as the reason for exclusion, this was because the record was not automatically excluded at an earlier screening stage due to inconsistent metadata between databases. * = correct citation as compared to original database record containing inaccuracies.

| Original ti ab classification | Title | Author(s) | Year | Reason for Exclusion |
| --- | --- | --- | --- | --- |
| Maybe | Treating disturbances in the relationship between mothers with bulimic eating disorders and their infants: a randomized, controlled trial of video feedback | Stein et al. | 2006 | No parent anxiety outcome |
| Maybe | Randomized controlled trial of the Circle of Security-Intensive intervention for mothers with postpartum depression: maternal unresolved attachment moderates changes in sensitivity | Muhlhan et al. | 2020 | No parent anxiety outcome |
| Maybe | Prolactin, a potential mediator of reduced social interactive behavior in newborn infants following maternal perinatal depressive symptoms | Zhang et al. | 2017 | No parent anxiety outcome |
| Maybe | Mother-infant interaction: effects of a home intervention and ongoing maternal drug use | Schuler et al. | 2010 | No parent anxiety outcome |
| Maybe | Effects of preventive family service coordination for parents with mental illnesses and their children, a RCT | Wansink et al. | 2015 | No parent anxiety outcome |
| Maybe | A randomized controlled trial of a mother-infant or toddler parenting program: demonstrating effectiveness in practice | Hayes et al. | 2008 | Incorrect population (parental mental health problems not recruitment focus) |
| Maybe | A controlled clinical treatment trial of interpersonal psychotherapy for depressed pregnant women at 3 New York City sites | Spinelli et al. | 2013 | No parent anxiety outcome |
| Maybe | Attachment and Affect between Mothers with Depression and their Children: Longitudinal Outcomes of Child Parent Psychotherapy | Guild et al.* | 2021 | No parent anxiety outcome |
| Maybe | Dissemination of an evidence-based prevention innovation for aggressive children living in culturally diverse, urban neighborhoods: the Early Risers effectiveness study | August et al. | 2003 | Incorrect population (parental mental health problems not recruitment focus) |
| Maybe | Role of home visiting in improving parenting and health in families at risk of abuse and neglect: Results of a multicentre randomised controlled trial and economic evaluation | Barlow et al. | 2007 | No parent anxiety outcome |
| Maybe | Long-term mother and child mental health effects of a population-based infant sleep intervention: Cluster-randomized, controlled trial | Hiscock et al.* | 2008 | No parent anxiety outcome  Incorrect population |
| Maybe | Parenting enhancement, interpersonal psychotherapy to reduce depression in low-income mothers of infants and toddlers: a randomized trial | Beeber et al. | 2013 | No parent anxiety outcome |
| Maybe | Specificity of preventative pediatric intervention effects in early infancy | Beeghly et al. | 1995 | Incorrect population  No parent anxiety outcome |
| Maybe | Can typical US home visits affect infant attachment? Preliminary findings from a randomized trial of Healthy Families Durham | Berlin et al. | 2017 | Incorrect population  No parent anxiety outcome |
| Maybe | Effects of a community health worker delivered intervention on maternal depressive symptoms in rural Tanzania | Bliznashka et al. | 2021 | No relevant infant outcome |
| Maybe | The efficacy of the Triple P-Positive Parenting Program in improving parenting and child behavior: a comparison with two other treatment conditions | Bodenmann et al. | 2008 | Incorrect population (child age too high) |
| Maybe | Toward a developmentally informed approach to parenting interventions: Seeking hidden effects | Brock & Kochanska | 2016 | Incorrect population  No parent anxiety outcome |
| Maybe | A Single-Session, Web-Based Parenting Intervention to Prevent Adolescent Depression and Anxiety Disorders: Randomized Controlled Trial | Cardamone-Breen et al. | 2018 | Incorrect population (child age too high) |
| Maybe | Bending the Curve: A Community-Based Behavioral Parent Training Model to Address ADHD-Related Concerns in the Voluntary Sector in Denmark | Chacko & Scavenius | 2018 | Incorrect population (child age too high) |
| Maybe | The effect of counseling with a skills training approach on maternal functioning: A randomized controlled clinical trial | Chamgurdani et al. | 2020 | No parent anxiety outcome |
| Maybe | Home Visiting and Antenatal Depression Affect the Quality of Mother and Child Interactions in South Africa | Christodoulou et al. | 2019 | No parent anxiety outcome |
| Maybe | Cognitive-behavioral depression treatment for mothers of children with attention-deficit/hyperactivity disorder. | Chronis et al. | 2006 | Incorrect population (child age too high) |
| Maybe | Development and preliminary evaluation of an integrated treatment targeting parenting and depressive symptoms in mothers of children with attention-deficit/hyperactivity disorder | Chronis-Tuscano et al. | 2013 | Incorrect population (child age too high) |
| Maybe | The efficacy of toddler-parent psychotherapy to increase attachment security in offspring of depressed mothers | Cicchetti et al. | 1999 | No parent anxiety outcome |
| Maybe | The efficacy of toddler-parent psychotherapy for fostering cognitive development in offspring | Cicchetti et al. | 2000 | No parent anxiety outcome |
| Maybe | Improving quality of mother-infant relationship and infant attachment in socioeconomically deprived community in South Africa: Randomised controlled trial | Cooper et al. | 2009 | Incorrect population  No parent anxiety outcome |
| Maybe | A RCT of peer-mentoring for first-time mothers in socially disadvantaged areas (the MOMENTS Study) | Cupples et al. | 2011 | Incorrect population  No parent anxiety outcome |
| Maybe | Emotional and cardiovascular reactivity to a child-focused interpersonal stressor among depressed mothers of psychiatrically ill children | Cyranowski et al. | 2009 | Incorrect population (child age too high) |
| Maybe | Chronic Maternal Depressive Symptoms Are Associated With Reduced Socio-Emotional Development in Children at 2 Years of Age: Analysis of Data From an Intervention Cohort in Rural Pakistan | De Oliveira et al. | 2019 | No parent anxiety outcome (parent anxiety measure too broad) |
| Maybe | Building Healthy Children: A preventive intervention for high-risk young families | Demeusy et al. | 2021 | No parent anxiety outcome |
| Maybe | Family connections: A program for preventing child neglect | DePanfilis & Dubowitz | 2005 | Incorrect population (child age too high)  No parent anxiety outcome |
| Maybe | Impact of behavioral feeding intervention on child emotional and behavioral functioning, maternal parenting stress, and mother-child relationships | Knight et al.* | 2019 | Incorrect population (parental mental health problems not recruitment focus, and child age too high) |
| Maybe | Effect of an early perinatal depression intervention on long-term child development outcomes: Follow-up of the Thinking Healthy Programme randomised controlled trial | Maselko et al.* | 2015 | No parent anxiety outcome |
| Maybe | Couple-Focused Prevention at the Transition to Parenthood, a Randomized Trial: Effects on Coparenting, Parenting, Family Violence, and Parent and Child Adjustment | Feinberg et al. | 2016 | Incorrect population (parental mental health problems not recruitment focus) |
| Maybe | Effects of family foundations on parents and children: 3.5 years after baseline | Feinberg et al. | 2010 | No parent anxiety outcome  Incorrect population (parental mental health problems not recruitment focus) |
| Maybe | Empirical Support for a Treatment Program for Families of Young Children With Externalizing Problems | Abbott-Feinfield & Baker | 2004 | Incorrect population (child age too high) |
| Maybe | Home visiting intervention for vulnerable families with newborns: follow-up results of a randomized controlled trial | Fraser et al. | 2000 | No parent anxiety outcome  Incorrect population (parental mental health problems not recruitment focus) |
| Maybe | Mental health promotion and prevention interventions in families with parental depression: A randomized controlled trial | Giannakopoulos et al. | 2021 | Incorrect population (child age too high) |
| Maybe | Behavioral Interventions for Infant Sleep Problems: A Randomized Controlled Trial | Gradisar et al. | 2016 | Incorrect population (parental mental health problems not recruitment focus) |
| Maybe | The impact of parent-delivered intervention on parents of very young children with autism | Estes et al.* | 2014 | No parent anxiety outcome (parent anxiety measure too broad) |
| Maybe | Feasibility and acceptability of an early home visit intervention aimed at supporting a positive mother-infant relationship for mothers at risk of postpartum depression | Greve et al. | 2018 | No parent anxiety outcome |
| Maybe | Relationships between parental sleep quality, fatigue, cognitions about infant sleep, and parental depression pre and post-intervention for infant behavioral sleep problems | Hall et al. | 2017 | No parent anxiety outcome |
| Maybe | Promoting Positive Mother-Infant Relationships: A Randomized Trial of Community Doula Support For Young Mothers | Hans et al. | 2013 | No parent anxiety outcome |
| Maybe | Informing Precision Home Visiting: Identifying Meaningful Subgroups of Families Who Benefit Most from Family Spirit | Haroz et al. | 2019 | No parent anxiety outcome |
| Maybe | Depressed mothers' neonates improve following the MABI and a Brazelton demonstration | Hart et al. | 1998 | No parent anxiety outcome |
| Maybe | Consistent use of bedtime parenting strategies mediates the effects of sleep education on child sleep: secondary findings from an early-life randomized controlled trial | Hatch et al. | 2019 | No parent anxiety outcome |
| Maybe | Randomised controlled trial of behavioural infant sleep intervention to improve infant sleep and maternal mood | Hiscock & Wake | 2002 | Incorrect population (parental mental health problems not recruitment focus) |
| Maybe | Long-term mother and child mental health effects of a population-based infant sleep intervention: cluster-randomized, controlled trial | Hiscock et al. | 2008 | Duplicate |
| Maybe | Universal parenting programme to prevent early childhood behavioural problems: Cluster randomised trial | Hiscock et al. | 2008 | Incorrect population (parental mental health problems not recruitment focus) |
| Maybe | Newborn Behavioral Observation, maternal stress, depressive symptoms and the mother-infant relationship: results from the Northern Babies Longitudinal Study (NorBaby) | Høifødt et al. | 2020 | Incorrect population (parental mental health problems not recruitment focus) |
| Maybe | Targeting genetic and environmental risk for mental illness in the womb | Hunter et al. | 2019 | Full text unavailable (symposium abstract only) |
| Maybe | Efficacy of learning through play plus intervention to reduce maternal depression in women with malnourished children: A randomized controlled trial from Pakistan | Husain et al. | 2021 | No parent anxiety outcome (parent anxiety measure too broad) |
| Maybe | Evaluating the Incredible Years Toddler Parenting Programme with parents of toddlers in disadvantaged (Flying Start) areas of Wales | Hutchings et al. | 2017 | Incorrect population (parental mental health problems not recruitment focus)  No parent anxiety outcome |
| Maybe | Improving parental stress levels among mothers living with HIV: A randomized control group intervention study | Johnson et al. | 2015 | Incorrect population (parental mental health problems not recruitment focus)  No parent anxiety outcome |
| Maybe | Supporting insensitive mothers: The Vilnius randomized control trial of video-feedback intervention to promote maternal sensitivity and infant attachment security | Kalinauskiene et al. | 2009 | Incorrect population (parental mental health problems not recruitment focus)  No parent anxiety outcome |
| Maybe | Clinical overview of children with mucopolysaccharidosis type III A and effect of Risperidone treatment on children and their mothers psychological status | Kalkan Ucar et al. | 2010 | Incorrect population (child age too high) |
| Maybe | Behavioral and socioemotional outcomes through age 5 years of the legacy for children public health approach to improving developmental outcomes among children born into poverty | Kaminski et al. | 2013 | Incorrect population (parental mental health problems not recruitment focus)  No parent anxiety outcome |
| Maybe | Evaluation of Lay Support in Pregnant women with Social risk (ELSIPS): a randomised controlled trial | Kenyon et al. | 2012 | Study protocol |
| Maybe | Is integrated private-clinic based early child development care effective? A clustered randomised trial in Pakistan | Khan et al. | 2018 | No parent anxiety outcome |
| Maybe | Exploring differences between adolescents and adults with perinatal depression—data from the Expanding Care for Perinatal Women With Depression Trial in Nigeria | Oladeji et al.* | 2019 | No parent anxiety outcome |
| Maybe | Effect of a maternal role training program on postpartum maternal role competence in nulliparous women with unplanned pregnancy | Kordi et al. | 2016 | Full text in Arabic |
| Maybe | The protective effects of father involvement for infants of teen mothers with depressive symptoms | Lewin et al. | 2015 | Incorrect population (parental mental health problems not recruitment focus)  No parent anxiety outcome |
| Maybe | Effect of a family intervention on psychological outcomes of children affected by parental HIV | Li et al. | 2014 | Incorrect population (child age too high; parental mental health problems not recruitment focus) |
| Maybe | Does maternal role functioning improve with antidepressant treatment in women with postpartum depression? | Logsdon et al. | 2009 | No parent anxiety outcome (parent anxiety measure too broad) |
| Maybe | The Efficacy of Using Peer Mentors to Improve Maternal and Infant Health Outcomes in Hispanic Families: Findings from a Randomized Clinical Trial | Lutenbacher et al. | 2018 | Incorrect population (parental mental health problems not recruitment focus)  No parent anxiety outcome |
| Maybe | What makes a difference: Early Head Start evaluation findings in a developmental context | Love et al. | 2013 | Full text unavailable (monograph abstract only) |
| Maybe | Home again: effects of the Mother-Child Home Program on mother and child | Madden et al. | 1984 | No parent anxiety outcome |
| Maybe | Improved child mental health following brief relationship enhancement and co-parenting interventions during the transition to parenthood | Tomfohr-Madsen et al.* | 2020 | Incorrect population (parental mental health problems not recruitment focus)  No parent anxiety outcome |
| Maybe | Maternal mood scores in mid-pregnancy are related to aspects of neonatal immune function | Mattes et al. | 2009 | No relevant infant outcome  No parent anxiety outcome |
| Maybe | Effectiveness of an Interpersonal Psychotherapy (IPT) Group Depression Treatment for Head Start Mothers: A Cluster-Randomized Controlled Trial | Mennen et al. | 2021 | Incorrect population (child age too high)  No parent anxiety outcome |
| Maybe | Does a perinatal parenting intervention work for fathers? A randomized controlled trial | Mihelic et al. | 2018 | Incorrect population (parental mental health problems not recruitment focus) |
| Maybe | The Effectiveness of an App-Based Nurse-Moderated Program for New Mothers With Depression and Parenting Problems (eMums Plus): Pragmatic Randomized Controlled Trial | Sawyer et al.* | 2019 | No parent anxiety outcome |
| Maybe | Maintaining stable parenting for young children through military life transitions | Mogil et al. | 2016 | Full text unavailable (presentation abstract only) |
| Maybe | Effect of a food supplementation and psychosocial stimulation trial for severely malnourished children on the level of maternal depressive symptoms in Bangladesh | Nahar et al. | 2015 | No parent anxiety outcome |
| Maybe | Mindfulness-based stress reduction for parents of young children with developmental delays: Implications for parental mental health and child behavior problems | Neece et al. | 2014 | Incorrect population (child age too high) |
| Maybe | Mitigating the effect of persistent postnatal depression on child outcomes through an intervention to treat depression and improve parenting: a randomised controlled trial | Stein et al. | 2018 | Duplicate |
| Maybe | The breathing bear: an intervention for crying babies and their mothers | Novosad et al. | 2003 | Incorrect population (parental mental health problems not recruitment focus)  No parent anxiety outcome |
| Maybe | An intervention to decrease uncertainly and distress among parents of children newly diagnosed with diabetes: A pilot study | Page et al. | 2005 | Incorrect population (child age too high; parental mental health problems not recruitment focus) |
| Maybe | Blended Infant Massage-Parenting Enhancement Program on Recovering Substance-Abusing Mothers' Parenting Stress, Self-Esteem, Depression, Maternal Attachment, and Mother-Infant Interaction | Porter et al. | 2015 | No parent anxiety outcome |
| Maybe | Impact of ‘‘controlled crying’’ on child and parent mental health to 6 years: randomised controlled trial. | Price et al. | 2010 | Full text unavailable (conference abstract only) |
| Maybe | Effects of an infant sleep intervention at child age 6 years: randomised controlled trial | Price et al. | 2011 | Full text unavailable (conference abstract only) |
| Maybe | Inconsolable infant crying and maternal postpartum depressive symptoms | Radesky et al. | 2013 | No parent anxiety outcome |
| Maybe | Adding "Circle of Security - Parenting" to treatment as usual in three Swedish infant mental health clinics. Effects on parents' internal representations and quality of parent-infant interaction | Risholm Mothander et al. | 2018 | No parent anxiety outcome (only available at baseline) |
| Maybe | Effects of home visits by paraprofessionals and by nurses: Age 4 follow-up results of a randomized trial | Olds et al.* | 2004 | No parent anxiety outcome |
| Maybe | The efficacy of toddler-parent psychotherapy to reorganize attachment in the young offspring of mothers with major depressive disorder: A randomized preventive trial | Toth et al.* | 2006 | No parent anxiety outcome (only available at baseline) |
| Maybe | Influence of relationship skills education on pathways of associations between paternal depressive symptoms and IPV and childhood behaviors | Roopnarine et al. | 2018 | No parent anxiety outcome |
| Maybe | A community-based randomized controlled trial of Mom Power parenting intervention for mothers with interpersonal trauma histories and their young children | Rosenblum et al. | 2017 | Incorrect population (parental mental health problems not recruitment focus) |
| Maybe | Improving Maternal Representations in High-Risk Mothers: A Randomized, Controlled Trial of the Mom Power Parenting Intervention | Rosenblum et al. | 2018 | Incorrect population (parental mental health problems not recruitment focus) |
| Maybe | 20.4 - Infant Mental Health Home Visiting Buffers the Adverse Impact of Maternal Adverse Childhood Experiences on Toddler and Parent Outcomes | Rosenblum et al. | 2020 | Full text unavailable (conference abstract only) |
| Maybe | A randomized controlled trial of mother-infant psychoanalytic treatment: II. Predictive and moderating influences of qualitative patient factors | Salomonsson et al. | 2011 | No parent anxiety outcome |
| Maybe | A Long-term follow-up of a randomized controlled trial of mother-infant psychoanalytic treatment: Outcomes on the children | Salomonsson et al. | 2015 | No parent anxiety outcome |
| Maybe | A long-term follow-up study of a randomized controlled trial of mother-infant psychoanalytic treatment: Outcomes on mothers and interactions | Salomonsson et al. | 2015 | No parent anxiety outcome |
| Maybe | The incredible years parents and babies program: A pilot randomized controlled trial | Pontoppidan et al.* | 2016 | Incorrect population (parental mental health problems not recruitment focus)  No parent anxiety outcome |
| Maybe | Effects of parental intervention on behavioural and psychological outcomes for Kurdish parents and their children | Sangawi et al. | 2018 | Incorrect population (child age too high) |
| Maybe | The Effectiveness of an App-Based Nurse-Moderated Program for New Mothers With Depression and Parenting Problems (eMums Plus): Pragmatic Randomized Controlled Trial | Sawyer et al. | 2019 | Duplicate |
| Maybe | Parent Management Training Oregon Model and Family-Based Services as Usual for Behavioral Problems in Youth: A National Randomized Controlled Trial in Denmark | Scavenius et al. | 2020 | Incorrect population (child age too high) |
| Maybe | Efficacy of the “Tuebinger-Intensiv-Programm fur Eltern” in Treating Childhood Anxieties - A Pilot Study | Schlarb et al. | 2015 | Incorrect population (child age too high) |
| Maybe | Prevention of behavior problems in a selected population: Stepping Stones Triple P for parents of young children with disabilities | Shapiro et al. | 2014 | Incorrect population (parental mental health problems not recruitment focus) |
| Maybe | Development and pilot evaluation of an Internet-facilitated cognitive-behavioral intervention for maternal depression | Sheeber et al. | 2012 | Incorrect population (child age too high)  No parent anxiety outcome |
| Maybe | A randomized-controlled trial to examine the effectiveness of the 'Home-but not Alone' mobile-health application educational programme on parental outcomes | Shorey et al. | 2017 | Incorrect population (parental mental health problems not recruitment focus)  No parent anxiety outcome |
| Maybe | Effectiveness of a Technology-Based Supportive Educational Parenting Program on Parental Outcomes (Part 1): Randomized Controlled Trial | Shorey et al. | 2019 | Incorrect population (parental mental health problems not recruitment focus) |
| Maybe | Multiple mediation analysis of the peer-delivered Thinking Healthy Programme for perinatal depression: Findings from two parallel, randomised controlled trials | Singla et al. | 2021 | No parent anxiety outcome |
| Maybe | New Beginnings for mothers and babies in prison: a cluster randomized controlled trial. | Sleed et al. | 2013 | Incorrect population (parental mental health problems not recruitment focus)  No parent anxiety outcome |
| Maybe | Effects of video feedback on early coercive parent-child interactions: the intervening role of caregivers' relational schemas | Smith et al. | 2013 | Incorrect population (parental mental health problems not recruitment focus)  No parent anxiety outcome |
| Maybe | Does improvement in maternal attachment representations predict greater maternal sensitivity, child attachment security and lower rates of relapse to substance use? A second test of Mothering from the Inside Out treatment mechanisms | Suchman et al. | 2018 | Incorrect population (child age too high)  No parent anxiety outcome |
| Maybe | Mothering from the Inside Out: results of a pilot study testing a mentalization-based therapy for mothers enrolled in mental health services. | Suchman et al. | 2016 | Incorrect population (child age too high)  No parent anxiety outcome (parent anxiety measure too broad) |
| Maybe | Effectiveness of attachment based STEEP TM intervention in a German high-risk sample | Suess et al. | 2016 | Incorrect population (parental mental health problems not recruitment focus)  No parent anxiety outcome |
| Maybe | Group psychoeducational program for mothers of children with high functional pervasive developmental disorders: A randomized controlled trial | Suzuki et al. | 2012 | Full text unavailable (conference abstract only) |
| Maybe | Opioid addiction and neonatal abstinence syndrome (NAS, also known as neonatal opioid withdrawal syndrome): Effect of drug use targeted psychotherapy (DUST) on cessation of other addictive drug use | Tabi et al. | 2019 | Full text unavailable (conference abstract only) |
| Maybe | Maternal sleep and depressive symptoms: links with infant Negative Affectivity | Tikotzky et al. | 2010 | No parent anxiety outcome |
| Maybe | The efficacy of toddler-parent psychotherapy to reorganize attachment in the young offspring of mothers with major depressive disorder: a randomized preventive trial | Toth et al. | 2006 | Duplicate |
| Maybe | Outcomes following an early parenting center residential parenting program | Treyvaud et al. | 2009 | Incorrect population (parental mental health problems not recruitment focus) |
| Maybe | In-patient psychiatric-psychotherapeutic treatment of mothers with a generalized anxiety disorder--does the co-admission of their children influence the treatment results? A prospective, controlled study | Tritt et al. | 2004 | Incorrect population (child age too high)  No relevant infant outcomes |
| Maybe | A randomized controlled trial of a home-visiting intervention aimed at preventing relationship problems in depressed mothers and their infants | van Doesum et al. | 2008 | No parent anxiety measure |
| Maybe | Maternal depression and child behaviour problems. Randomised placebo-controlled trial of a cognitive-behavioural group intervention | Verduyn et al. | 2003 | Incorrect population (child age too high) |
| Maybe | Strengthening Attachment Competencies in Parents with Mental Illness: Adaptation and Pilot Testing of the Mentalization-Based Lighthouse Parenting Program | Volkert et al. | 2019 | No parent anxiety measure (Parent anxiety measure neither continuous nor categorical; it is qualitative data) |
| Maybe | Emotional Disclosure Through Journal Writing: Telehealth Intervention for Maternal Stress and Mother-Child Relationships | Whitney & Smith | 2015 | No parent anxiety measure |
| Maybe | A pilot study of a parent-education group for families affected by depression | Williams et al. | 2003 | Incorrect population (child age too high) |
| Maybe | Interruption of dysfunctional mother-child reciprocal influences associated with family therapy | Wu & Slesnick | 2019 | Incorrect population (child age too high) |
| Maybe | Intervening with Attachment and Biobehavioral Catch-Up to decrease disrupted parenting behavior and attachment disorganization: The role of parental withdrawal | Yarger et al. | 2020 | No parent anxiety measure |
| Maybe | Placentophagy's effects on mood, bonding, and fatigue: A pilot trial, part 2 | Young et al. | 2018 | Incorrect population (parental mental health problems not recruitment focus) |
| Maybe | Parenting skills and emotional availability: An RCT | Yousafzai et al. | 2015 | Incorrect population (parental mental health problems not recruitment focus)  No parent anxiety outcome (parent anxiety measure too broad) |
| Maybe | Co-Occurring Trajectory of Mothers' Substance Use and Psychological Control and Children's Behavior Problems: The Effects of a Family Systems Intervention | Zhang et al. | 2018 | Incorrect population (child age too high) |
| Maybe | Clinical Demonstration of the Potential of Parental Feedback in Reducing Deterioration During Group Psychotherapy With Children | Bitan et al.* | 2020 | Incorrect population (child age too high) |
| Maybe | Postpartum Depression Prevention through the Mother-Infant Dyad: The Role of Childhood Trauma | Berry et al. | 2021 | Combines data from Werner et al. (2016; included) and Scorza et al. (2020; parental mental health problems not recruitment focus) |
| Include | A randomized, controlled trial of nurse home visiting to vulnerable families with newborns | Armstrong et al. | 1999 | No parent anxiety outcome |
| Include | Paraprofessional-delivered home-visiting intervention for American Indian teen mothers and children: 3-year outcomes from a randomized controlled trial | Barlow et al. | 2015 | Incorrect population (parental mental health problems not recruitment focus) |
| Include | Parenting and early development among children of drug-abusing women: effects of home intervention | Black et al. | 1994 | No parent anxiety outcome |
| Include | Links between Shared Reading and Play, Parent Psychosocial Functioning, and Child Behavior: Evidence from a Randomized Controlled Trial | Canfield et al. | 2019 | Incorrect population (parental mental health problems not recruitment focus)  No parent anxiety outcome |
| Include | Mums 4 Mums: structured telephone peer-support for women experiencing postnatal depression. Pilot and exploratory RCT of its clinical and cost effectiveness | Caramlau et al. | 2011 | Study protocol |
| Include | Interactions and attachment in infants of mothers with OCD | Challacombe et al. | 2015 | Full text unavailable (conference abstract only) |
| Include | Attempting to prevent postnatal depression by targeting the mother-infant relationship: a randomised controlled trial | Cooper et al. | 2015 | No parent anxiety outcome^[[1]](#footnote-1)^ |
| Include | Maternal depression trajectories in adolescent mothers living in a poor urban area and their association with parental stress, infant behavioral problems, and psychological violence | Fatori et al. | 2017 | Incorrect population (parental mental health problems not recruitment focus) |
| Include | Establishing family foundations: intervention effects on coparenting, parent/infant well-being, and parent-child relations. | Feinberg et al. | 2008 | Incorrect population (parental mental health problems not recruitment focus) |
| Include | Establishing family foundations: intervention effects on coparenting, parent/infant well-being, and parent-child relations | Feinberg et al. | 2008 | Duplicate |
| Include | Randomized controlled trial of parent-infant psychotherapy for parents with mental health problems and young infants | Fonagy et al. | 2016 | No parent anxiety outcome |
| Include | Effective treatment for postpartum depression is not sufficient to improve the developing mother-child relationship | Forman et al. | 2007 | No parent anxiety outcome |
| Include | The efficacy of parent training for promoting positive parent-toddler relationships. | Gross et al. | 1995 | No parent anxiety outcome |
| Include | Long-term effects of a home-visiting intervention for depressed mothers and their infants | Kersten-Alvarez et al. | 2010 | No parent anxiety outcome |
| Include | Treatment of severe fear of childbirth with haptotherapy: Design of a multicenter randomized controlled trial | Klabbers et al. | 2014 | Study protocol |
| Include | Parent-Child Interaction Therapy with Toddlers: A Community-based Randomized Controlled Trial with Children Aged 14-24 Months | Kohlhoff et al. | 2020 | No parent anxiety outcome |
| Include | Effect of home-based peer support on maternal-infant interactions among women with postpartum depression: A randomized, controlled trial. | Letourneau et al. | 2011 | No parent anxiety outcome |
| Include | Video feedback compared to treatment as usual in families with parent-child interactions problems: A randomized controlled trial | Lydersen et al. | 2015 | No parent anxiety outcome |
| Include | Effectiveness of a peer-delivered, psychosocial intervention on maternal depression and child development at 3 years postnatal: a cluster randomised trial in Pakistan. | Maselko et al. | 2020 | No parent anxiety outcome |
| Include | A randomised controlled trial on intranasal oxytocin as an adjunct to interaction coaching to improve maternal bonding in women with mild postpartum depression | McErlean et al. | 2011 | Full text unavailable (conference abstract only)^[[2]](#footnote-2)^ |
| Include | Outcomes of a Randomized Trial of a Cognitive Behavioral Enhancement to Address Maternal Distress in Home Visited Mothers | McFarlane et al. | 2017 | No parent anxiety outcome |
| Include | Cumulative environmental risk in substance abusing women: early intervention, parenting stress, child abuse potential and child development. | Nair et al. | 2003 | No parent anxiety outcome^[[3]](#footnote-3)^ |
| Include | Infant outcomes following treatment of antenatal depression: Findings from a pilot randomized controlled trial | Netsi et al. | 2015 | No parent anxiety outcome |
| Include | Effects of lay support for pregnant women with social risk factors on infant development and maternal psychological health at 12 months postpartum. | Popo et al. | 2017 | No parent anxiety outcome |
| Include | Maternal patterns of antenatal and postnatal depressed mood and the impact on child health at 3-years postpartum. | Rotheram-Fuller et al. | 2018 | No parent anxiety outcome |
| Include | A randomized controlled trial of mother-infant psychoanalytic treatment: I. Outcomes on self-report questionnaires and external ratings | Salomonsson & Rolf | 2011 | No parent anxiety outcome |
| Include | Oxytocin in postnatally depressed mothers: Its influence on mood and expressed emotion | Mah et al. | 2013 | No parent anxiety outcome |
| Include | Controlled clinical trial of interpersonal psychotherapy versus parenting education program for depressed pregnant women. | Spinelli et al. | 2003 | No parent anxiety outcome |
| Include | A pilot randomised controlled trial to evaluate the feasibility and acceptability of the Baby Triple P Positive Parenting Programme in mothers with postnatal depression. | Tsivos et al. | 2015 | No parent anxiety outcome |
| Include | The effects of a music and singing intervention during pregnancy on maternal well-being and mother-infant bonding: a randomised, controlled study | Wulff et al. | 2021 | No parent anxiety outcome |
| Hand sought | Lay support for pregnant women with social risk: a randomised controlled trial | Kenyon et al. | 2016 | No parent anxiety outcome |
| Hand sought | What makes a difference: Early Head Start evaluation findings in a developmental context: III. Impacts of Early Head Start participation on child and parent outcomes at ages 2, 3, and 5 | Vogel et al. | 2013 | No parent anxiety outcome |
| Hand sought | A Trauma-Informed, Family-Centered, Virtual Home Visiting Program for Young Children: One-Year Outcomes | Mogil et al. | 2021 | Incorrect population (child age too high) |
| Hand sought | Outcomes at six years of age for children with infant sleep problems: longitudinal community-based study | Price et al. | 2012 | Incorrect population (parental mental health problems not recruitment focus) |
| Hand sought | A failure to confirm the effectiveness of a brief group psychoeducational program for mothers of children with high-functioning pervasive developmental disorders: a randomized controlled pilot trial | Suzuki et al. | 2014 | Incorrect population (child age too high) |
| Hand sought | Opioid addiction/pregnancy and neonatal abstinence syndrome (NAS): A preliminary open-label study of buprenorphine maintenance and drug use targeted psychotherapy (DUST) on cessation of addictive drug use. | Tabi et al. | 2020 | No parent anxiety outcome |
| Hand sought | Maternal Parenting Electronic Diary in the Context of a Home Visit Intervention for Adolescent Mothers in an Urban Deprived Area of São Paulo, Brazil: Randomized Controlled Trial | Fatori et al. | 2020 | Incorrect population (parental mental health problems not recruitment focus) |
| Hand sought | Haptotherapy as a new intervention for treating fear of childbirth: a randomized controlled trial. Journal of Psychosomatic Obstetrics & Gynecology, 40(1), 38-47. | Klabbers et al. | 2019 | No infant outcome |
| Hand sought | Preventing maternal mental health disorders in the context of poverty: pilot efficacy of a dyadic intervention | Scorza et al. | 2020 | Incorrect population (parental mental health problems not recruitment focus) |
| Key expert suggestion | Changes in infant emotion regulation following maternal cognitive behavioral therapy for postpartum depression | Krzeczkowski et al. | 2021 | No parent anxiety outcome (baseline co-morbid anxiety available only) |
| Hand sought | Effectiveness of a psycho-educational intervention for expecting parents to prevent postpartum parenting stress, depression and anxiety: a randomized controlled trial | Missler et al. | 2020 | Incorrect population (parental mental health problems not recruitment focus) |
| Hand sought | A randomized controlled trial of ‘MUMentum Pregnancy’: Internet-delivered cognitive behavioral therapy program for antenatal anxiety and depression | Loughnan et al. | 2019 | No relevant infant outcome (fetal rather than infant outcomes) |
| Maybe | Teaching attachment behaviors to pregnant women: a randomized controlled trial of effects on infant mental health from birth to the age of three months | Akbarzadeh et al. | 2017 | Unreliable reporting (discrepancies regarding main findings and nature of intervention, among other inconsistencies) |
| Hand sought | Relationship and mother-infant bonding outcomes following a psychological intervention for antenatal anxiety | Thompson-Booth | 2017 | Unpublished thesis without peer review |

## Table S7 Reasons for inclusion for articles assessed at full-text

| Original ti ab classification | Title | Author(s) | Year | Parent outcome measure (pre/post intervention) | Infant/parent-infant outcome measure (pre/post intervention) | All other eligibility criteria met (Y/N) |
| --- | --- | --- | --- | --- | --- | --- |
| Maybe | PREPP: postpartum depression prevention through the mother-infant dyad | Werner et al. | 2016 | Hamilton Anxiety Rating Scale (HAM-A; Hamilton, 1959) | Average daily frequency of fuss/cry episode; Baby’s Day Diary (Barr et al., 1988) | Yes |
| Include | Effects of psychological treatment of mental health problems in pregnant women to protect their offspring: Randomised controlled trial | Burger et al. | 2020 | Brief State-Trait Anxiety Inventory (Brief STAI; Marteau & Bekker, 1992) | CBCL (Internalising/Externalising; Rescorla, 2005); and Postpartum Bonding Questionnaire (PBQ; Brockington et al., 2006) | Yes |
| Include | A pilot randomized controlled trial of time-intensive cognitive-behaviour therapy for postpartum obsessive-compulsive disorder: effects on maternal symptoms, mother-infant interactions and attachment. | Challacombe et al. | 2017 | Yale–Brown Obsessive–Compulsive Scale (YBOCS; Goodman et al., 1989) | Numerous (x8 measures) including: Bates Infant Temperament Questionnaire (ITQ; Bates et al., 1979) and Ainsworth sensitivity/intrusiveness measures (Ainsworth et al., 1978) | Yes |
| Include | A therapeutic playgroup for depressed mothers and their infants: feasibility study and pilot randomized trial of community HUGS | Ericksen et al.* | 2018 | Depression, Anxiety and Stress Scale (DASS; Lovibond & Lovibond, 1995) | Paediatric Infant Parent Exam (PIPE; Fiese et al., 2001) | Yes |
| Hand sought | Feasibility study and pilot randomised trial of an antenatal depression treatment with infant follow-up | Milgrom et al.* | 2015 | Beck Anxiety Inventory (BAI; Beck & Steer, 1991) | Numerous, including: Ages and Stages Questionnaire Social Emotional (ASQ:SE: Squires et al., 2002) and Infant Behaviour Questionnaire Short Form (IBQ- R: Gartstein and Rothbart, 2003; Putnam et al., 2014) | Yes |
| Maybe | Improving the mother-infant relationship following postnatal depression: a randomised controlled trial of a brief intervention (HUGS) | Holt et al.* | 2021 | Beck Anxiety Inventory (BAI; Beck & Steer, 1991) | Numerous, including: ASQ-SE (Squires et al., 2002); PBQ (Brockington et al., 2006) | Yes |
| Include | Perinatal Dyadic Psychotherapy for postpartum depression: a randomized controlled pilot trial | Goodman et al. | 2015 | State-Trait Anxiety Inventory (STAI; Spielberger et al., 1970)  Anxiety disorder measured by the Structured Clinical Interview for DSM-IV-R for Axis I disorders (SCID-IV-R; First et al., 1998) | Coding Interactive Behavior manual (CIB; Feldman, 1998): maternal sensitivity, dyadic reciprocity, infant involvement | Yes |
| Include | Lessons learned from a pilot randomized controlled trial of dyadic interpersonal psychotherapy for perinatal depression in a low-income population | Lenze et al. | 2020 | Brief State-Trait Anxiety Inventory, State Scale (Berg et al., 1998) | Numerous including: the Infant-Toddler Social and Emotional Assessment (ITSEA; Carter et al., 1999); Coding Interactive Behavior manual (CIB; Feldman, 1998) | Yes |
| Maybe | Postnatal depression and mother and infant outcomes after infant massage | O'Higgins et al. | 2008 | Spielberger State Anxiety Inventory (SSAI; Spielberger et al., 1970) | Numerous including: Global Ratings for Mother–Infant Interactions (see Murray et al., 1996) | Yes |
| Hand sought | Netmums: a phase II randomized controlled trial of a guided Internet behavioural activation treatment for postpartum depression | O'Mahen et al. | 2014 | Generalised Anxiety Disorder 7-item screening tool (GAD-7; Spitzer et al., 2006) | Postnatal Bonding Questionnaire (PBQ; Brockington et al., 2006) | Yes |
| Maybe | Mitigating the effect of persistent postnatal depression on child outcomes through an intervention to treat depression and improve parenting: a randomised controlled trial. | Stein et al. | 2018 | Generalised Anxiety Disorder (and Posttraumatic Stress Disorder) as measured by the Structured Clinical Interview for DSM-IV-R for Axis I disorders (SCID-IV-R; First et al., 1998) | Numerous including CBCL and child emotion-regulation assessed with the barrier paradigm from the Laboratory Temperament Assessment Battery (Lab-TAB; Goldsmith & Rothbart, 1996) | Yes |
| Hand sought | An exploratory parallel-group randomised controlled trial of antenatal Guided Self-Help (plus usual care) versus usual care alone for pregnant women with depression: DAWN trial | Trevillion et al. | 2020 | Proportion meeting Generalized Anxiety Disorder-7 (GAD-7; Spitzer et al., 2006) criteria for anxiety (i.e., score of ≥8) | Postnatal Bonding Questionnaire (PBQ; Brockington et al., 2006) | Yes |

## 1 Intervening for perinatal anxiety *v.* intervening for broad risk or transdiagnostic symptoms

Before beginning a review of interventions for perinatal anxiety, it was necessary to consider what population should be within scope. Perinatal anxiety, like the umbrella term ‘anxiety,’ may be operationalised in myriad ways. It can refer to an observable response (state anxiety) or a propensity towards anxiety (trait anxiety; Reiss, 1997; Spielberger, 1985). It may refer to a range of cut-offs on a dimensional scale (‘severe’, ‘moderate’, ‘mild’), or a series of conditions classified in a diagnostic manual (‘generalised anxiety disorder’, ‘social anxiety disorder’, ‘panic disorder’). Some cognitive and physiological features of anxiety are thought to be common to other psychiatric diagnoses (Faustino, 2021; Grisanzio et al., 2018), and there is further overlap between the constructs of stress and anxiety (though stress is considered more specific and less diffuse; Epel et al., 2018). Perinatal anxiety risk is also likely to increase in the context of a broad range of socio-economic circumstances and environmental stressors (Furtado et al., 2018; Leach et al., 2017), though over half of individuals who go on to later develop mental health or developmental conditions do not have identifiable risk factors (Hiscock et al., 2008; Offord et al., 1998). In the present review, we took a clinical, rather than risk-based or universal approach to determining the scope of the population. We recognised both dimensional and categorical conceptualisations of perinatal anxiety, as well as a broad range of anxiety diagnoses. We also acknowledged that perinatal anxiety would often be studied alongside other co-occurring conditions (especially depression).

## 2 Risk of bias assessments

An overview of the results from the risk of bias assessments is presented in Figure 2 of the main text. The majority of studies were at low risk of bias arising from the randomisation process - perhaps due to standard reporting guidelines, which state randomisation methods must be detailed (Schulz et al., 2010). In addition, most studies were at low risk of bias with respect to missing data. This was mainly due to low rates of attrition, or as a result of sensitivity analyses that were able to demonstrate that results were little changed under a range of plausible assumptions about the relationship between missingness in the outcome and its true value. There was one major exception to this: where lack of detail regarding missing outcome data and information available about the trial context led to a judgement of high risk (Werner et al., 2016).

With regard to bias arising from deviations from the intended intervention, risk levels were mixed. Five studies were judged to be low risk due to consistency between the intended intervention detailed in trial protocols/registry records and the final intervention reported (Ericksen et al., 2018; Goodman et al., 2015; Holt et al., 2021; Lenze et al., 2020; Milgrom et al., 2015). Any inconsistencies that were identified were justified by the authors (e.g., Milgrom et al., 2015). The remaining studies were judged to be of some concern due to inconsistencies between the intended interventions and the final interventions reported, or inadequate detail about the intended interventions (Burger et al., 2020; Challacombe et al., 2017; O’Higgins et al., 2008; O’Mahen et al., 2014; Stein et al., 2018; Trevillion et al., 2020; Werner et al., 2016). Where it was clear that deviations had occurred, these were either balanced between the intervention and control groups (Stein et al., 2018; Werner et al., 2016) or were unlikely to affect the outcome of interest (Burger et al., 2020; Trevillion et al., 2020).

With regard to bias in outcome measurement, risk levels varied again. Four studies were judged to be low risk due to the outcome assessor being masked to participants’ group allocation (Challacombe et al., 2017; Goodman et al., 2015; Holt et al., 2021; O’Higgins et al., 2008). Seven studies were judged to be of some concern due to the outcome being measured by participant-report, despite participants being unmasked to group allocation (Burger et al., 2020; Ericksen et al., 2018; Milgrom et al., 2015; O’Mahen et al., 2014; Stein et al., 2018; Trevillion et al., 2020; Werner et al., 2016). While outcome assessment could have been influenced by knowledge of the intervention received, this was not thought to be likely due to the participants’ low probability of ‘therapy allegiance.^[[4]](#footnote-4)^ For one study, it was not clear from the information provided whether the research team responsible for outcome assessment were masked to group allocation (Lenze et al., 2020). If unmasked, it is possible the research team held some degree of allegiance to the trial intervention that would bias their outcome assessment (Dragioti et al., 2015). However, such researcher allegiance was not reported. Consequently, a judgement of ‘some concern’ was made.

Concern was most substantive with regard to bias in the selection of the reported result. This was because half of the studies did not provide adequate detail on the intended analyses of the trial, either as a result of not registering their trials (Challacombe et al., 2017; O’Higgins et al., 2008; O’Mahen et al., 2014), or as a result of limited detail within available trial registry records (Ericksen et al., 2018) or the trial protocol (Trevillion et al., 2020). Five studies were also judged to be of some concern in this domain. This was either due to intended analyses being partly misaligned with the final analyses reported (Burger et al., 2020; Stein et al., 2018) or indicative of internal consistency despite a lack of adequate detail regarding the intended analyses (Goodman et al., 2015; Lenze et al., 2020). Two were judged to be high risk, due to a timepoint reporting discrepancy that indicated potential selectivity (Holt et al., 2021; Werner et al., 2016).

Note that one specific outcome measure and numerical result were used for the risk of bias assessments, for which the rationale is described below.

# Bias arising from wait-list or treatment as usual control conditions

The above bias assessments do not account for bias arising from the design of the control condition. Research has shown that less specific control conditions (e.g., wait-list or treatment as usual groups) amplify the apparent efficacy of the intervention group. Placebo controls outperform wait-list groups, and provide benefit, such that the effect size of a cognitive behavioural therapy (CBT) intervention is halved by comparing it to a placebo rather than a wait-list group (Zhu et al., 2014). There is also evidence that the effect size of all psychotherapies for depression drops by 19% by removing wait-list controlled studies (Cuijpers et al., 2018). This is worth noting, as at least half of the studies in the present review used a treatment as usual or wait-list control condition (Burger et al., 2020; Challacombe et al., 2017; Ericksen et al., 2018; Goodman et al., 2015; Milgrom et al., 2015; Trevillion et al., 2020).

# Statistical power limitations

The above bias assessments also do not account for the studies’ statistical power. As insufficient power can lead to inflated effect sizes (Button et al., 2013; Ioannidis, 2008), this information is important for interpreting results.

Half of the studies within this review were pilot studies (Challacombe et al., 2017; Ericksen et al., 2018; Goodman et al., 2015; Lenze et al., 2020; Milgrom et al., 2015; Werner et al., 2016). Of these, one study met the intended sample size identified through power calculations (based on the primary outcome measure of maternal mood; Milgrom et al., 2015), while another did not (Ericksen et al., 2018). The remaining four studies did not include formal power calculations, prohibiting an assessment of statistical power (Challacombe et al., 2017; Goodman et al., 2015; Lenze et al., 2020; Werner et al., 2016). Most of these four studies directly acknowledged the potential for underpowered analyses, particularly in relation to assessment of between group differences (Goodman et al., 2015; Lenze et al., 2020; Werner et al., 2016).

The remaining six studies of this review represent mixed levels of statistical power. Three studies were adequately powered, meeting the intended sample size identified through power calculations (Burger et al., 2020; O’Mahen et al., 2014; Stein et al., 2018). Power calculations by Burger et al. (2020) were based on an outcome measure relevant to this review – that is, a measure of infant socio-emotional development - whereas Stein et al. (2018) and O’Mahen et al. (2014) were not. By contrast, two studies conducted analyses based on smaller than intended sample sizes, resulting in potentially insufficient power (Holt et al., 2021; Trevillion et al., 2020). One study made no mention of power calculations, prohibiting assessment of statistical power (O’Higgins et al., 2008).

## Rationale for RoB assessment: numerical result selection

The Risk of Bias (RoB) 2 tool requires that bias assessments be conducted on a specific outcome measure and numerical result (Sterne et al., 2019). Wherever possible, we selected a significant between group effect as the result on which to base the RoBs. We selected an infant or dyadic outcome given the project’s principal orientation towards child development. We did not elect to conduct RoB assessments on parent anxiety measures as not all studies calculated inferential statistics on parent anxiety outcome measures (e.g., Stein et al., 2018). Only participants belonging to groups that were randomised were considered in the RoB assessments. We prioritised between group effects as these are most likely to detect differences in the outcome according to the intervention (rather than, for instance, effects of study engagement or the ‘dodo bird’ effect; Enck & Zipfel, 2019). We prioritised significant over non-significant results as positive findings are more likely to be biased than null findings (Sterne et al., 2019). Preference was then given to primary over secondary outcome results; if multiple results were significant, we made our selection from these results at random. Note that for Lenze et al. (2020), a within group effect was selected as no between group analyses were conducted. For Goodman et al. (2015), a non-significant between group effect was selected rather than a significant within group effect.

|  | Small | Medium | Large |
| --- | --- | --- | --- |
| Hedges *g/*Cohen’s *d* | ~ .2 | .3-.7 | >.8 |
| Odds Ratios | ~ 1.5 | 1.6-3 | > 5 |

## Table S8 An approximate guide to interpreting the strengths of associations represented by Hedges *g/*Cohen’s *d*, as well as odds ratios (Chen et al., 2010).

## 3 Interventions demonstrating deteriorations in outcome measures

Two studies identified statistically significant, medium sized effects indexing deterioration in relevant outcome measures (Burger et al., 2020; Ericksen et al., 2018).

Burger et al. (2020), using the Brief State-Trait Anxiety Inventory (Brief STAI; Marteau & Bekker, 1992), noted a significant, medium sized treatment effect on anxiety symptoms during the intervention at 24 weeks gestation, such that anxiety scores were higher in the intervention group (*g* = 0.4). This effect disappeared thereafter. The trial authors suggested this result was related to the exposure component of the intervention, which involved approaching fear-provoking stimuli as a means of overcoming avoidance behaviour and its unintended consequences (Burger et al., 2020). The authors also performed a subgroup analysis on mothers meeting diagnostic criteria for anxiety disorders; this showed a significant result for birth outcomes, such that infants’ gestational ages were lower in the intervention group compared with the control condition if they had anxious parents. The trial authors speculated that the increased anxiety effect at 24 weeks gestation may have been correlated with increased physiological arousal, in turn adversely affecting intrauterine development of the fetus (Burger et al., 2020). This outcome is not strictly within scope of the present review – however, the potential link to parent anxiety renders it of interest. It is also worth highlighting that, post-intervention, ratings of anxiety remained slightly elevated in the intervention group compared to the control. Child internalising and externalising scores also remained slightly elevated in the intervention group compared to the control at 18 months postpartum. These results represented small, non-significant effects (Burger et al., 2020).

Ericksen et al. (2018) also identified adverse treatment side-effects. The authors, using the Parenting Stress Index Short Form (PSI-SF; Abidin, 1995), found that average scores on the ‘difficult child’ subscale remained higher in the intervention group compared to the control condition. The difficult child subscale includes 12 items probing both elements of infant temperament (e.g., ‘My child gets upset easily over the smallest thing’) and the impact of this on the parent (e.g., ‘My child makes more demands on me than most children’; PSI-SF; Abidin, 1995). This medium sized effect (*g* = 0.3) remained significant after adjustment for baseline imbalances between groups. There were also small, directional deteriorations on the ‘parent-infant dysfunctional behaviour’ subscale, and in parent-infant interaction, the latter assessed via the Paediatric Infant Parent Exam (Fiese et al., 2001). Neither of these reached significance, and these analyses were underpowered.

## 4 Component-by-component breakdown of adult-focused interventions

Of the five adult-focused interventions, three were delivered postnatally (Challacombe et al., 2017; O’Mahen et al., 2014) while three were delivered prenatally (Burger et al., 2020; Milgrom et al., 2015; Trevillion et al., 2020). All interventions were delivered via individual rather than group sessions. Interventions evaluated by Trevillion et al. (2020) and O’Mahen et al. (2014) used a CBT-based, guided self-help model, i.e., low-intensity treatment involving the support of a health professional to guide the use of a self-help manual or e-resource (Coull & Morris, 2011). One intervention was delivered via an intensive model whereby hours were compressed into a relatively short treatment period (Challacombe et al., 2017).

All of the interventions in this grouping incorporated techniques from CBT. Three interventions included CBT strategies for anxiety-related conditions, including elements such as exposure, response prevention, and/or cognitive-restructuring (Burger et al., 2020; Challacombe et al., 2017; O’Mahen et al., 2014). This involves controlled exposure to fear-provoking stimuli, resisting ‘escape behaviour’ when in anxiety-inducing situations, and reappraising ‘maladaptive’ thoughts and beliefs that maintain psychological distress (Bolton & Perrin, 2008; Clark, 2013). Three interventions also included CBT strategies for mood-related conditions, such as depression; for example, cognitive-restructuring and problem-solving exercises (Burger et al., 2020; Milgrom et al., 2015; Trevillion et al., 2020). Two studies also explicitly focused on or emphasised the use of behavioural activation (Burger et al., 2020; O’Mahen et al., 2014). Behavioural activation draws on behavioural aspects of CBT while omitting its cognitive features (Ekers et al., 2014). It draws on the principles of operant conditioning and other functional analytical approaches, and has been found to be as effective as standard CBT for managing depression (Richards et al., 2016). One study used CBT techniques specific to PTSD (Burger et al., 2020).

Independent of the CBT strategies, three studies in this grouping incorporated intervention components related to supporting participants with: (a) establishing a more healthy lifestyle and (b) managing their social networks to maximise support available (Milgrom et al., 2015; O’Mahen et al., 2014; Trevillion et al., 2020). Milgrom et al. (2015) also included a component related to relaxation training. In addition to the adult-directed components, some of the studies in this grouping also incorporated a small number of components relating more specifically to the infant or dyad (see components 1-10; Table 3 of main text).

## 5 Component-by-component breakdown of infant or dyad-focused interventions

Of the seven infant or dyad-focused interventions, all were delivered predominantly postnatally, with one exception that split a roughly equal number of sessions over the prenatal and postnatal period (Lenze et al., 2020). Four interventions were delivered using individual sessions (including the infant; Goodman et al., 2015; Lenze et al., 2020; Stein et al., 2018; Werner et al., 2016), while three operated via group format (Ericksen et al., 2018; Holt et al., 2021; O’Higgins et al., 2008).

Six out of seven of the interventions in this grouping incorporated elements of interaction coaching, including support with how to read, understand or respond to infant cues (exception: Werner et al., 2016). Three interventions included an attachment-based exploration of the parent-infant relationship (Ericksen et al., 2018; Lenze et al., 2020; Stein et al., 2018), while three provided information on infant temperament or developmental stages (Ericksen et al., 2018; Goodman et al., 2015; Lenze et al., 2020). Four interventions incorporated therapeutic approaches examining the parent’s patterns of relating to others, including, for example, how the mother’s own memories of childhood or representation of her child informs the dyadic relationship (Goodman et al., 2015; Holt et al., 2021; Lenze et al., 2020; Werner et al., 2016). Two interventions incorporated elements of play therapy or sensory activities (Ericksen et al., 2018; Holt et al., 2021), and three incorporated infant massage (Ericksen et al., 2018; Holt et al., 2021; O’Higgins et al., 2008). One intervention explicitly conceptualised the infant as a psychological agent (Stein et al., 2018), one provided practical support focused on infant behaviours such as fussing, feeding and sleeping (Werner et al., 2016), one included ‘good enough’ parenting principles (Holt et al., 2021), and one provided explicit support with the transition to parenthood (Goodman et al., 2015).

Of note, two of the infant-focused interventions included a prefatory CBT programme before the ‘main’ intervention; however, both intervention and active control groups attended the CBT programme, reducing the potential to detect CBT-specific between group effects (Holt et al., 2021; Stein et al., 2018).

# References

Barr, R. G., Kramer, M. S., Boisjoly, C., McVey-White, L., & Pless, I. B. (1988). Parental diary of infant cry and fuss behaviour. *Archives of Disease in Childhood*, *63*(4), 380–387. https://doi.org/10.1136/adc.63.4.380

Bolton, D., & Perrin, S. (2008). Evaluation of exposure with response-prevention for obsessive compulsive disorder in childhood and adolescence. *Journal of Behavior Therapy and Experimental Psychiatry*, *39*(1), 11–22. https://doi.org/10.1016/j.jbtep.2006.11.002

Burger, H., Verbeek, T., Aris-Meijer, J. L., Beijers, C., Mol, B. W., Hollon, S. D., Ormel, J., Pampus, M. G. van, & Bockting, C. L. H. (2020). Effects of psychological treatment of mental health problems in pregnant women to protect their offspring: Randomised controlled trial. *The British Journal of Psychiatry*, *216*(4), 182–188. https://doi.org/10.1192/bjp.2019.260

Button, K. S., Ioannidis, J. P. A., Mokrysz, C., Nosek, B. A., Flint, J., Robinson, E. S. J., & Munafò, M. R. (2013). Power failure: Why small sample size undermines the reliability of neuroscience. *Nature Reviews Neuroscience*, *14*(5), 365–376. https://doi.org/10.1038/nrn3475

Challacombe, F. L., Salkovskis, P. M., Woolgar, M., Wilkinson, E. L., Read, J., & Acheson, R. (2017). A pilot randomized controlled trial of time-intensive cognitive–behaviour therapy for postpartum obsessive–compulsive disorder: Effects on maternal symptoms, mother–infant interactions and attachment. *Psychological Medicine*, *47*(8), 1478–1488. https://doi.org/10.1017/S0033291716003573

Chen, H., Cohen, P., & Chen, S. (2010). How Big is a Big Odds Ratio? Interpreting the Magnitudes of Odds Ratios in Epidemiological Studies. *Communications in Statistics - Simulation and Computation*, *39*(4), 860–864. https://doi.org/10.1080/03610911003650383

Clark, D. A. (2013). Cognitive Restructuring. In *The Wiley Handbook of Cognitive Behavioral Therapy* (pp. 1–22). American Cancer Society. https://doi.org/10.1002/9781118528563.wbcbt02

Coull, G., & Morris, P. G. (2011). The clinical effectiveness of CBT-based guided self-help interventions for anxiety and depressive disorders: A systematic review. *Psychological Medicine*, *41*(11), 2239–2252. https://doi.org/10.1017/S0033291711000900

Cuijpers, P., Karyotaki, E., Reijnders, M., & Ebert, D. D. (2018). Was Eysenck right after all? A reassessment of the effects of psychotherapy for adult depression. *Epidemiology and Psychiatric Sciences*, 1–10.

Dragioti, E., Dimoliatis, I., Fountoulakis, K. N., & Evangelou, E. (2015). A systematic appraisal of allegiance effect in randomized controlled trials of psychotherapy. *Annals of General Psychiatry*, *14*(1), 25. https://doi.org/10.1186/s12991-015-0063-1

Enck, P., & Zipfel, S. (2019). Placebo Effects in Psychotherapy: A Framework. *Frontiers in Psychiatry*, *0*. https://doi.org/10.3389/fpsyt.2019.00456

Epel, E. S., Crosswell, A. D., Mayer, S. E., Prather, A. A., Slavich, G. M., Puterman, E., & Mendes, W. B. (2018). More than a feeling: A unified view of stress measurement for population science. *Frontiers in Neuroendocrinology*, *49*, 146–169. https://doi.org/10.1016/j.yfrne.2018.03.001

Ericksen, J., Loughlin, E., Holt, C., Rose, N., Hartley, E., Buultjens, M., Gemmill, A. W., & Milgrom, J. (2018). A Therapeutic Playgroup for Depressed Mothers and Their Infants: Feasibility Study and Pilot Randomized Trial of Community Hugs. *Infant Mental Health Journal*, *39*(4), 396–409. https://doi.org/10.1002/imhj.21723

Faustino, B. (2021). Transdiagnostic perspective on psychological inflexibility and emotional dysregulation. *Behavioural and Cognitive Psychotherapy*, *49*(2), 233–246. https://doi.org/10.1017/S1352465820000600

Furtado, M., Chow, C. H. T., Owais, S., Frey, B. N., & Van Lieshout, R. J. (2018). Risk factors of new onset anxiety and anxiety exacerbation in the perinatal period: A systematic review and meta-analysis. *Journal of Affective Disorders*, *238*, 626–635. https://doi.org/10.1016/j.jad.2018.05.073

Goodman, J. H., Prager, J., Goldstein, R., & Freeman, M. (2015). Perinatal Dyadic Psychotherapy for postpartum depression: A randomized controlled pilot trial. *Archives of Women’s Mental Health*, *18*(3), 493–506. https://doi.org/10.1007/s00737-014-0483-y

Grisanzio, K. A., Goldstein-Piekarski, A. N., Wang, M. Y., Rashed Ahmed, A. P., Samara, Z., & Williams, L. M. (2018). Transdiagnostic Symptom Clusters and Associations With Brain, Behavior, and Daily Function in Mood, Anxiety, and Trauma Disorders. *JAMA Psychiatry*, *75*(2), 201–209. https://doi.org/10.1001/jamapsychiatry.2017.3951

Hiscock, H., Bayer, J. K., Price, A., Wake, M., Ukoumunne, O. C., & Rogers, S. (2008). Universal parenting programme to prevent early childhood behavioural problems: Cluster randomised trial. *BMJ*, *336*(7639), 318–321. https://doi.org/10.1136/bmj.39451.609676.AE

Holt, C., Gentilleau, C., Gemmill, A. W., & Milgrom, J. (2021). Improving the mother-infant relationship following postnatal depression: A randomised controlled trial of a brief intervention (HUGS). *Archives of Women’s Mental Health*. https://doi.org/10.1007/s00737-021-01116-5

Ioannidis, J. P. A. (2008). Why Most Discovered True Associations Are Inflated. *Epidemiology*, *19*(5), 640–648.

Leach, L. S., Poyser, C., & Fairweather‐Schmidt, K. (2017). Maternal perinatal anxiety: A review of prevalence and correlates. *Clinical Psychologist*, *21*(1), 4–19. https://doi.org/10.1111/cp.12058

Lenze, S. N., Potts, M. A., Rodgers, J., & Luby, J. (2020). Lessons learned from a pilot randomized controlled trial of dyadic interpersonal psychotherapy for perinatal depression in a low-income population. *Journal of Affective Disorders*, *271*, 286–292.

Milgrom, J., Holt, C., Holt, C. J., Ross, J., Ericksen, J., & Gemmill, A. W. (2015). Feasibility study and pilot randomised trial of an antenatal depression treatment with infant follow-up. *Archives of Women’s Mental Health*, *18*(5), 717–730. https://doi.org/10.1007/s00737-015-0512-5

Offord, D. R., Kraemer, H. C., Kazdin, A. E., Jensen, P. S., & Harrington, R. (1998). Lowering the Burden of Suffering From Child Psychiatric Disorder: Trade‐Offs Among Clinical, Targeted, and Universal Interventions. *Journal of the American Academy of Child & Adolescent Psychiatry*, *37*(7), 686–694. https://doi.org/10.1097/00004583-199807000-00007

O’Higgins, M., St James Roberts, I., & Glover, V. (2008). Postnatal depression and mother and infant outcomes after infant massage. *Journal of Affective Disorders*, *109*(1–2), 189–192.

O’Mahen, H. A., Richards, D. A., Woodford, J., Wilkinson, E., McGinley, J., Taylor, R. S., & Warren, F. C. (2014). Netmums: A phase II randomized controlled trial of a guided Internet behavioural activation treatment for postpartum depression. *Psychological Medicine*, *44*(8), 1675–1689. https://doi.org/10.1017/S0033291713002092

Reiss, S. (1997). Trait anxiety: It’s not what you think it is. *Journal of Anxiety Disorders*, *11*(2), 201–214. https://doi.org/10.1016/S0887-6185(97)00006-6

Schulz, K. F., Altman, D. G., Moher, D., & the CONSORT Group. (2010). CONSORT 2010 Statement: Updated guidelines for reporting parallel group randomised trials. *Trials*, *11*(1), 32. https://doi.org/10.1186/1745-6215-11-32

Spielberger, C. D. (1985). Assessment of state and trait anxiety: Conceptual and methodological issues. *Southern Psychologist*, *2*(4), 6–16.

Stein, A., Netsi, E., Lawrence, P. J., Granger, C., Kempton, C., Craske, M. G., Nickless, A., Mollison, J., Stewart, D. A., Rapa, E., West, V., Scerif, G., Cooper, P. J., & Murray, L. (2018). Mitigating the effect of persistent postnatal depression on child outcomes through an intervention to treat depression and improve parenting: A randomised controlled trial. *The Lancet Psychiatry*, *5*(2), 134–144.

Sterne, J. A. C., Savović, J., Page, M. J., Elbers, R. G., Blencowe, N. S., Boutron, I., Cates, C. J., Cheng, H.-Y., Corbett, M. S., Eldridge, S. M., Emberson, J. R., Hernán, M. A., Hopewell, S., Hróbjartsson, A., Junqueira, D. R., Jüni, P., Kirkham, J. J., Lasserson, T., Li, T., … Higgins, J. P. T. (2019). RoB 2: A revised tool for assessing risk of bias in randomised trials. *BMJ*, *366*, l4898. https://doi.org/10.1136/bmj.l4898

Trevillion, K., Ryan, E. G., Pickles, A., Heslin, M., Byford, S., Nath, S., Bick, D., Milgrom, J., Mycroft, R., Domoney, J., Pariante, C., Hunter, M. S., & Howard, L. M. (2020). An exploratory parallel-group randomised controlled trial of antenatal Guided Self-Help (plus usual care) versus usual care alone for pregnant women with depression: DAWN trial. *Journal of Affective Disorders*, *261*, 187–197. https://doi.org/10.1016/j.jad.2019.10.013

Werner, E. A., Gustafsson, H. C., Lee, S., Feng, T., Jiang, N., Desai, P., & Monk, C. (2016). PREPP: postpartum depression prevention through the mother-infant dyad. *Archives of Women’s Mental Health*, *19*(2), 229–242. https://doi.org/10.1007/s00737-015-0549-5

Zhu, Z., Zhang, L., Jiang, J., Li, W., Cao, X., Zhou, Z., Zhang, T., & Li, C. (2014). Comparison of psychological placebo and waiting list control conditions in the assessment of cognitive behavioral therapy for the treatment of generalized anxiety disorder: A meta-analysis. *Shanghai Archives of Psychiatry*, *26*(6), 319–331. https://doi.org/10.11919/j.issn.1002-0829.214173

1. Anxiety during pregnancy is reported at baseline but not mentioned thereafter; no pre/post parent anxiety outcome measure. [↑](#footnote-ref-1)
2. Could not identify full report of this study so contacted the authors and received following response: ‘Due to having null findings the first author didn’t pursue publication. So unfortunately it’s a file drawer null effect.’ (Prof Mark Dadds; personal communication; 10 June, 2021). [↑](#footnote-ref-2)
3. The Brief Symptom Inventory (BSI; Derogatis & Melisaratos, 1983) probes anxiety as one of nine psychological wellbeing dimensions at baseline, but this is not reported and there is no post-intervention anxiety measure. [↑](#footnote-ref-3)
4. Typically, therapy allegiance occurs among those familiar with a specific treatment, such as researchers or therapists. Individuals who have not previously received therapy are unlikely to be partial to one or another type of treatment (Dragioti et al., 2015). [↑](#footnote-ref-4)
